# Cytokine-induced autophagy promotes long-term VCAM-1 but not ICAM-1 expression by degrading late-phase IκBα

**DOI:** 10.1038/s41598-017-12641-8

**Published:** 2017-09-29

**Authors:** Ling-Yun Chu, Ying-Chang Hsueh, Hsiao-Ling Cheng, Kenneth K. Wu

**Affiliations:** 1 Metabolomic Medicine Research Center, China Medical University Hospital, Taichung, Taiwan; 2 Graduate Institute of Biomedical Sciences, China Medical University, Taichung, Taiwan; 3 Institute of Cellular and System Medicine, National Health Research Institutes, Zhunan, Taiwan; 4 Department of Medical Sciences, National Tsing-Hua University, Hsinchu, Taiwan

## Abstract

Pro-inflammatory cytokines are known to induce endothelial cell autophagy, but the role of autophagy in regulating the expression of pro-inflammatory molecules has not been characterized. We hypothesized that autophagy facilitates expression of endothelial adhesion molecules. TNFα and IL-1β induced autophagy markers in human umbilical vein endothelial cells and inhibition of autophagy by 3-methyladenine (3-MA) blocked adhesion of Jurkat lymphocytes. Interestingly, 3-MA suppressed VCAM-1 but not ICAM-1 expression at 24 hours but not 6 hours. 3-MA suppressed VCAM-1 transcription and decreased nuclear NF-κB p65 level at 6 hours but not at 2 hours. Cytokines induced a biphasic degradation of IκBα and 3-MA selectively blocked the late-phase IκBα degradation. Our results suggest that cytokine-induced autophagy contributes to late-phase IκBα degradation, facilitates NF-κB nuclear translocation and VCAM-1 transcription for long-term VCAM-1 expression. With a cytokines array assay, we found that 3-MA also inhibited IP-10 expression. These findings provide new information about the role of endothelial autophagy in persistent expression of VCAM-1 and IP-10 which enhance lymphocyte recruitment and adhesion to endothelium.

## Introduction

Pro-inflammatory mediators such as tumor necrosis factor α (TNFα) disrupt endothelial barrier function and induce expression of endothelial surface adhesion molecules leading to vascular and tissue inflammation and damage^1,2^. TNFα stimulates transcription of vascular cellular adhesion molecule-1 (VCAM-1) and intercellular adhesion molecule-1 (ICAM-1) via which it increases VCAM-1 and ICAM-1 expression and induces leukocyte adhesion to endothelial cell surface and transmigration through endothelium^3,4^. It has been reported that TNFα induces endothelial adhesion molecule expression through activation of NF-κB^3,5^. NF-κB comprises Rel (Rel A, C-Rel, Rel B) and NF-κB (p100, p105) family proteins which form hetero- or homodimers to activate gene expression. p65 (Rel A) and p50 (derived from p105) heterodimers are the key NF-κB mediating pro-inflammatory mediator-induced gene expression^6^. Under basal cellular condition, p65 is bound by IκBs notably IκBα and sequestered in the cytoplasm. Upon stimulation by pro-inflammatory cytokines, IκBα is phosphorylated by IκB kinases (IKK) and degraded via ubiquitin-proteasome system resulting in liberation of p65^7^. p65 and p50 are translocated to nucleus where they bind to specific motifs on promoters and activate diverse genes including ICAM-1 and VCAM-1^8^. Recent reports suggest that IκBα is degraded via autophagy and lysosomal degradation^9^. IκBα degradation was reported to be biphasic. Upon cellular stimulation IκBα is rapidly degraded within minutes but is resynthesized and its level is restored at 1-2 h. The resynthesized IκBα undergoes a late-phase (at approximately 6 h after cellular stimulation) degradation. Both phases of IκBα degradation is generally considered to be processed via ubiquitin-proteasomes.

Autophagy is a conserved cellular process which degrades organelles, protein aggregates and fat complexes and recycles the degradation products to maintain cellular homeostasis during nutritional deprivation^10,11^. Autophagy has been shown to play important roles in maintaining normal vascular function by regulating macrophage lipid metabolism, shear-stress-induced vascular nitric oxide, angiogenesis and von Willebrand factor biosynthesis^12,13^. Pro-inflammatory mediators such as TNFα were reported to induce autophagy in diverse cell types^14–16^. However, it remains to be determined whether TNFα-induced autophagy is involved in biphasic IκBα degradation, nor is it clear whether autophagy regulates TNFα-induced persistent VCAM-1 and ICAM-1 expression in endothelial cells. Here, we propose that TNFα- and IL-1β-induced autophagy is involved in late-phase IκBα degradation and thereby maintains long-term adhesion molecules expression in endothelial cells stimulated by pro-inflammatory cytokines. To test this hypothesis, we investigated TNFα- and IL-1β-induced autophagy in human umbilical vein endothelial cells (HUVECs) and determined its impact on IκB degradation, VCAM-1/ICAM-1 expression, cytokines release, and lymphocyte adhesion. Our results indicate that cytokine-induced autophagy mediates the late-phase IκBα degradation, thereby maintaining long-term VCAM-1 and IP-10 expression and facilitating lymphocyte adhesion to endothelial cells.

## Results

### Cytokines induce autophagy via an mTOR-independent mechanism to facilitate lymphocytes adhesion to endothelial cells

**Figure 1:**
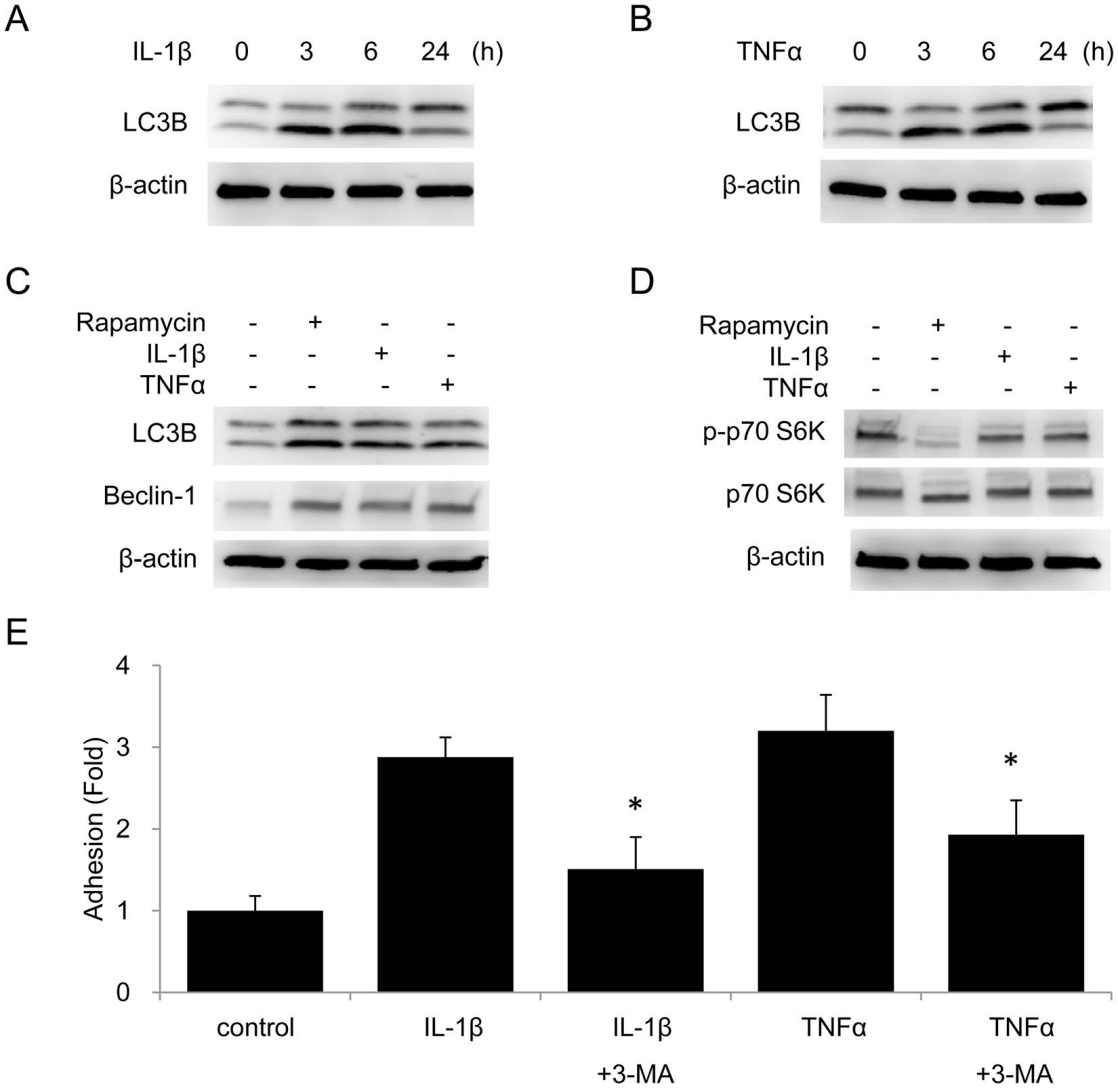
Pro-inflammatory cytokines induce autophagy which facilitates Jurkat cells adhesion to HUVECs. (**A**,**B**) HUVECs were treated with 10 ng/mL IL-1β (**A**) or 10 ng/mL TNFα (**B**) for indicated time. LC3B was analyzed by western blotting. (**C**,**D**) HUVECs were treated with 100 nmol/L rapamycin, 10 ng/mL IL-1β, or 10 ng/mL TNFα for 6 h. LC3B and Beclin-1 (**C**) and phospho-S6K and S6K (**D**) were analyzed by western blotting. (**E**) HUVECs were treated with 10 ng/mL IL-1β or 10 ng/mL TNFα with or without 5 mmol/L 3-MA for 24 h. Leukotracker-labeled Jurkat cells were then added onto HUVECs for 1 h. Adhesion of Jurkat cells was determined by the fluorescence at 480 nm/520 nm. Each bar denotes mean ± SD of three independent experiments. *Indicates P < 0.05 vs. IL-1β or TNFα alone.

As the effect of TNFα or IL-1β on endothelial cell autophagy has not been extensively evaluated, we analyzed LC3B-II formation in HUVECs treated with TNFα or IL-1β. IL-1β induced LC3B-II in a time-dependent manner; LC3B-II formation reached maximal at 6 h and returned to basal level at 24 h (Fig. 1A). TNFα induced LC3B-II following a similar time course as IL-1β (Fig. 1B). Rapamycin is known to induce autophagy by inhibiting mTOR complex. Using rapamycin treatment as a reference for comparison, TNFα and IL-1β induced LC3B-II formation and increased Beclin-1 level to an extent comparable to rapamycin (Fig. 1C). By contrast, neither IL-1β nor TNFα inhibited phosphorylation of p70 S6K, a downstream signaling of mTOR (Fig. 1D), which indicates that IL-1β and TNFα induce autophagy via a mechanism independent to mTOR inhibition. To determine whether cytokine-induced autophagy plays a role in regulating endothelial cell interaction with lymphocytes, we examined adhesion of Jurkat cells to cytokine-activated HUVECs. Adhesion of Jurkat cells to HUVECs was tested 24 h after IL-1β or TNFα stimulation with or without 3-MA, an autophagy inhibitor. As shown in Fig. 1E, IL-1β and TNFα increased Jurkat cells adhesion to HUVECs, which was significantly inhibited by 3-MA. These results suggest that cytokines induce autophagy via an mTOR-independent manner and cytokine-induced autophagy facilitates lymphocytes adhesion to activated endothelial cells.

### Inhibition of autophagy selectively inhibits VCAM-1 expression but not ICAM-1 expression

**Figure 2:**
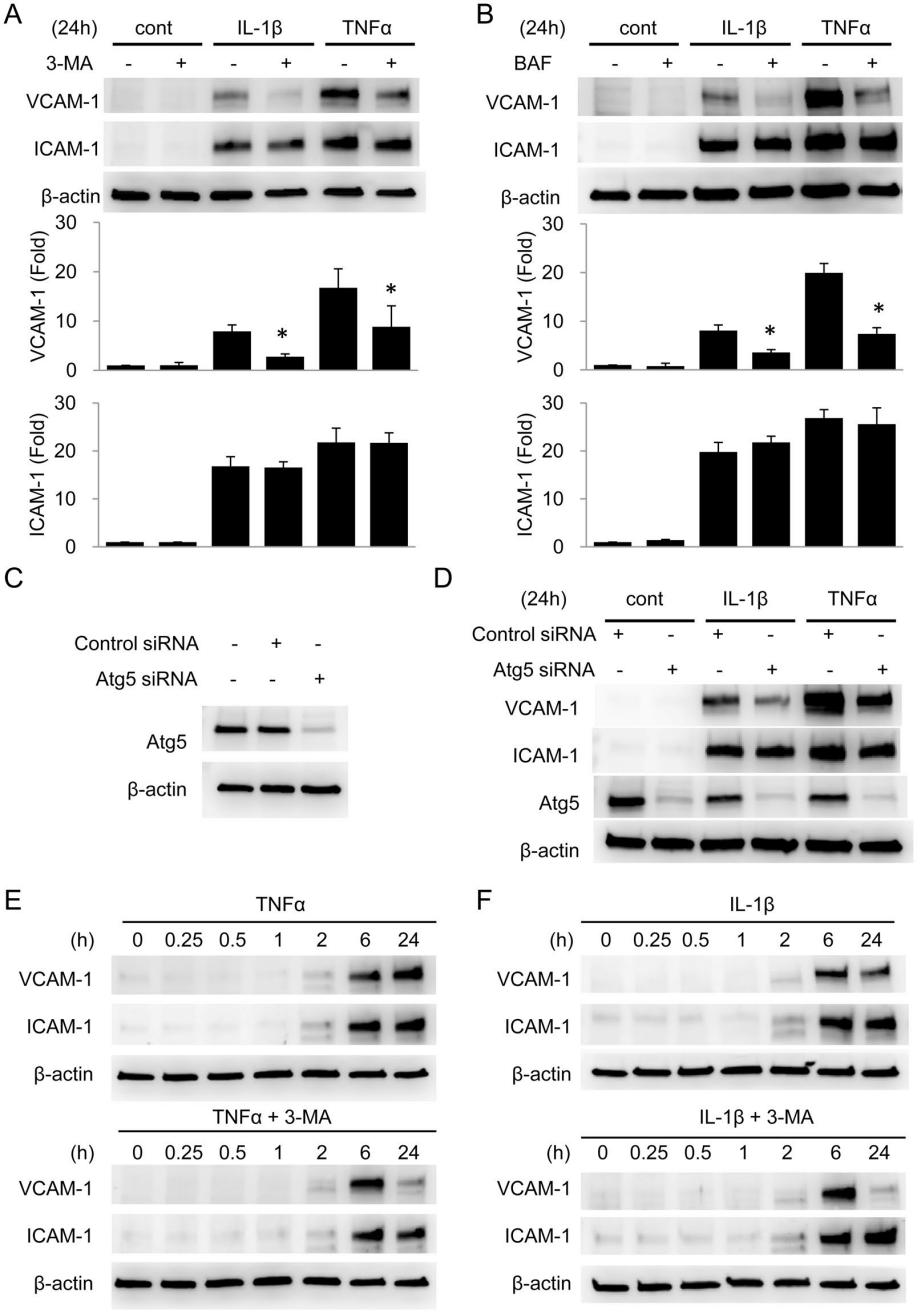
Autophagy facilitates long-term (24 h) expression of VCAM-1 in HUVECs. (**A**,**B**) HUVECs were pretreated with or without 5 mmol/L 3-MA (**A**) or 100 nmol/L Bafilomycin A (BAF) (**B**) for 30 min followed by 10 ng/mL IL-1β or 10 ng/mL TNFα for 24 h. VCAM-1 and ICAM-1 were analyzed by western blotting. Upper panel shows a representative blot and the lower panel the quantitative analysis. Each bar denotes mean ± SD of three independent experiments. *Indicates P < 0.05. (**C**) HUVECs were transfected with Atg5 siRNA or control siRNA for 72 h. Atg5 was analyzed by western blotting. (**D**) HUVECs were transfected with siRNA for 72 h followed by 10 ng/mL IL-1β or 10 ng/mL TNFα for 24 h. VCAM-1, ICAM-1 and Atg5 were analyzed by western blotting. (**E**,**F**) HUVECs were pretreated with or without 5 mmol/L 3-MA followed by 10 ng/mL TNFα (**E**) or 10 ng/mL IL-1β (**F**) for indicated time. VCAM-1 and ICAM-1 were analyzed by western blotting.

Leukocytes adhesion to endothelium is mediated by interaction of leukocyte integrins with cytokine-induced endothelial adhesion molecules such as VCAM-1 and ICAM-1. To test whether autophagy regulates cytokine-induced expression of VCAM-1 and/or ICAM-1 in endothelial cells, we pretreated HUVECs with 3-MA for 30 min followed by IL-1β or TNFα for 24 h. Although 3-MA had no effect on ICAM-1 expression, it inhibited IL-1β- and TNFα-induced VCAM-1 expression (Fig. 2A). To confirm the effect of autophagy inhibition on VCAM-1 expression, we pretreated HUVECs with Bafilomycin A (BAF), an inhibitor of autolysosomal degradation, for 30 min followed by IL-1β or TNFα for 24 h. BAF exerted a similar effect as 3-MA, in which it inhibited only VCAM-1 expression (Fig. 2B). To further confirm that autophagy is required for VCAM-1 expression, we blocked autophagy with Atg5 siRNA transfection. Expression of Atg5 in HUVECs was reduced to ~20% of control by siRNA transfection (Fig. 2C). Transfection of Atg5 siRNA suppressed IL-1β- and TNFα-induced VCAM-1 expression at 24 h (Fig. 2D). Time-course experiments revealed that 3-MA only inhibited TNFα-induced VCAM-1 expression at 24 h but not at 6 h and had no effect on ICAM-1 expression at 6 h or 24 h (Fig. 2E). It inhibited IL-1β-induced VCAM-1 expression in a similar time-dependent manner and had no effect on ICAM-1 expression (Fig. 2F). Taken together, these results suggest that autophagy activation is selectively regulated for TNFα- and IL-1β-induced VCAM-1 expression at 24 h but not at 6 h.

### 3-MA inhibits VCAM-1 expression in a MAPK independent manner

**Figure 3:**
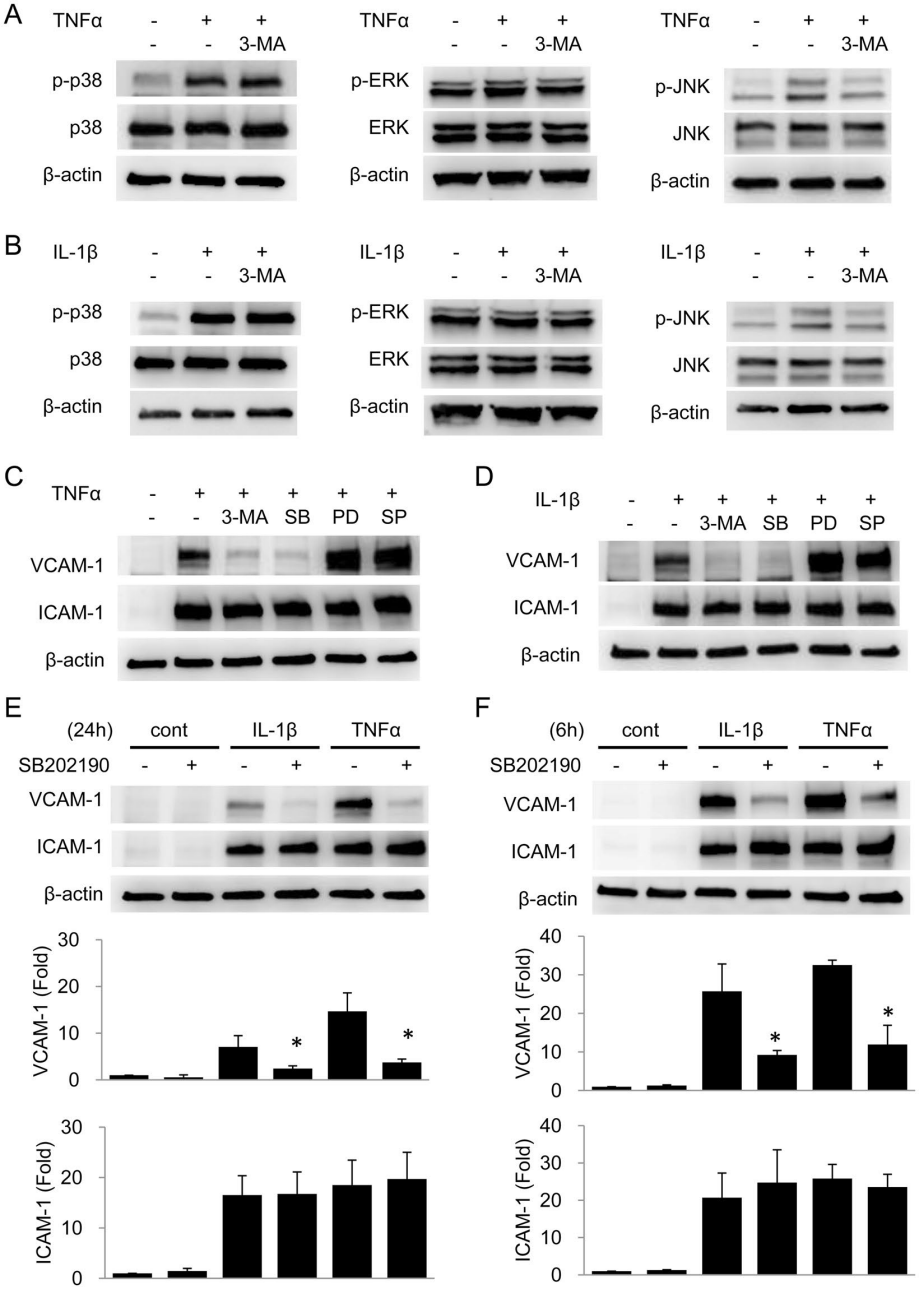
Autophagy facilitates VCAM-1 expression via a MAPK-independent mechanism. (**A**,**B**) HUVECs were pretreated with or without 5 mmol/L 3-MA for 30 min followed by 10 ng/mL TNFα (**A**) or 10 ng/mL IL-1β (**B**) for 5 min. Phosphorylated p38, ERK1/2 or JNK and total MAPK proteins were analyzed by western blotting. (**C**,**D**) HUVECs were pretreated with 5 mmol/L 3-MA, 10 μmol/L SB202190 (SB), 10 μmol/L PD98059 (PD) or 10 μmol/L SP600125 (SP) followed by 10 ng/mL TNFα (**C**) or 10 ng/mL IL-1β (**D**) for 24 h. VCAM-1 and ICAM-1 were analyzed by western blotting. (**E** and **F**) HUVECs were pretreated with or without 10 μmol/L SB202190 for 30 min followed by 10 ng/mL IL-1β or 10 ng/mL TNFα for 24 h (**E**) or 6 h (**F**). VCAM-1 and ICAM-1 were analyzed by western blotting. Upper panel shows a representative blot and the lower panel the quantitative analysis. Each bar denotes mean ± SD of three independent experiments. *Indicates P < 0.05.

It has been reported that 3-MA at high concentrations (>10 mmol/L) inhibits p38 MAPK or JNK^17^ and VCAM-1 expression is regulated by MAPKs^18,19^. Although the concentration of 3-MA we used is relatively low (5 mmol/L), we wondered whether 3-MA inhibits VCAM-1 expression via inhibiting MAPKs signaling. As shown in Fig. 3A, 3-MA at 5 mmol/L did not inhibit TNFα-induced p38 or ERK phosphorylation but inhibited TNFα-induced JNK phosphorylation. The effect of 3-MA on IL-1β-induced MAPK signaling was similar (Fig. 3B). To further examine whether 3-MA inhibits VCAM-1 expression via inhibiting MAPKs signaling, we compared the effect of 3-MA with that of SB202190, PD98059 and SP600125, which inhibit p38, ERK and JNK, respectively. While SB202190 inhibited TNFα- or IL-1β-induced VCAM-1 expression in a manner similar to 3-MA at 24 h, PD98059 and SP600125 increased VCAM-1 expression (Fig. 3C,D). To clarify whether 3-MA and SB202190 exert an identical effect on VCAM-1 expression, we determined long-term (24 h) and short-term (6 h) VCAM-1 expression in HUVECs treated with SB202190. SB202190 inhibited TNFα- and IL-1β-induced VCAM-1 expression at 24 h as well as 3-MA (Fig. 3E). However, it also inhibited VCAM-1 expression at 6 h (Fig. 3F). These results suggest that SB202190 inhibits VCAM-1 expression via a mechanism different from 3-MA and 3-MA inhibits VCAM-1 expression in a MAPK independent manner.

### Inhibition of autophagy reduces TNFα- and IL-1β-induced VCAM-1 transcription

**Figure 4:**
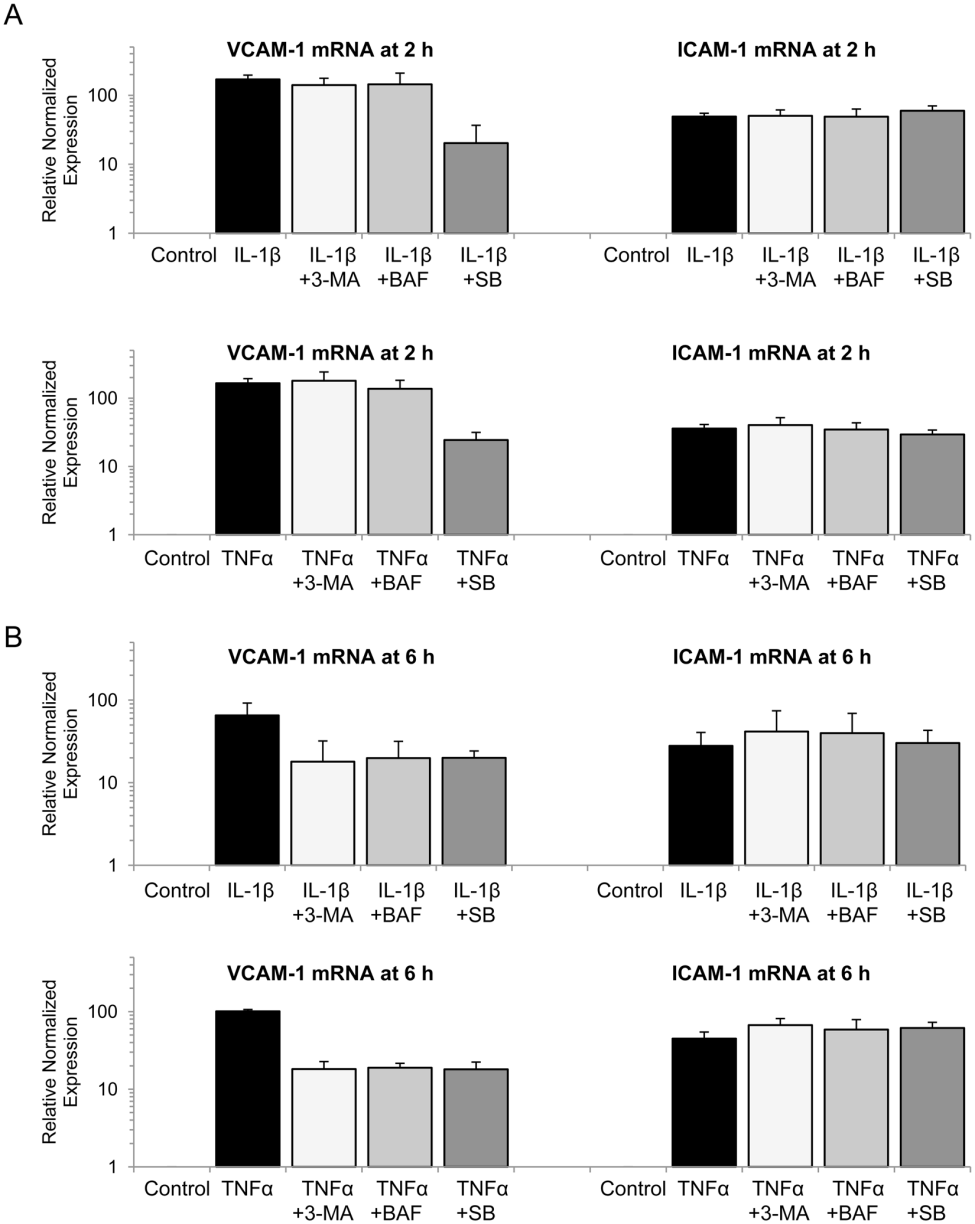
3-MA inhibits cytokines-induced VCAM-1 transcripts at 6 h but not at 2 h. (**A**,**B**) HUVECs were pretreated with 5 mmol/L 3-MA, 100 nmol/L Bafilomycin A (BAF) or 10 μmol/L SB202190 (SB) for 30 min followed by 10 ng/mL IL-1β or 10 ng/mL TNFα for 2 h (**A**) or 6 h (**B**). VCAM-1 and ICAM-1 mRNAs were quantified by qPCR using peptidylprolyl isomerase A (PPIA) as reference. Each bar denotes mean ± SD of two independent experiments.

We next evaluated the effect of 3-MA and BAF on VCAM-1 and ICAM-1 mRNA expression. HUVECs were pretreated with inhibitors for 30 min followed by TNFα or IL-1β for 2 h. VCAM-1 and ICAM-1 transcripts were analyzed by qPCR using PPIA as reference. As shown in Fig. 4A, TNFα and IL-1β significantly induced VCAM-1 and ICAM-1 transcripts at 2 h. Neither 3-MA nor BAF reduced VCAM-1 or ICAM-1 transcripts at 2 h, while SB202190 significantly inhibited transcription of VCAM-1 but not ICAM-1. This result is consistent with the protein expression (Fig. 3E) in that SB202190 inhibited early VCAM-1 but not ICAM-1 expression. As we observed that autophagy inhibitors blocked only late VCAM-1 expression (Fig. 2E,F), we evaluated the effect of 3-MA and BAF on VCAM-1 and ICAM-1 transcripts at a later time point. As shown in Fig. 4B, 3-MA and BAF significantly inhibited TNFα- or IL-1β-induced VCAM-1 transcripts as well as SB202190 at 6 h, but did not affect ICAM-1 transcripts. These results support the notion that autophagy facilitates VCAM-1 expression only at a late time point.

### Autophagy facilitates NF-κB p65 nuclear translocation after cytokines stimulation

**Figure 5:**
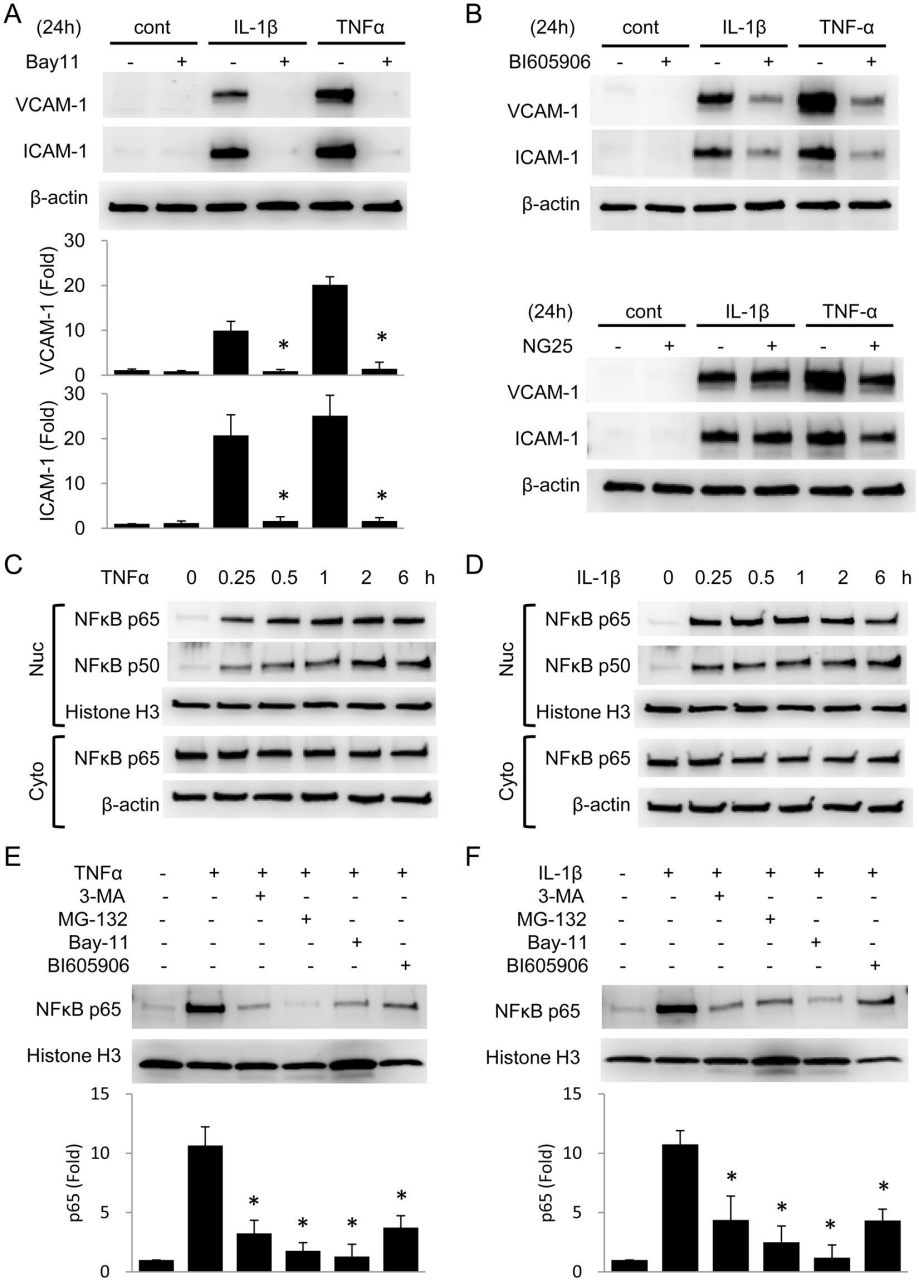
3-MA inhibits cytokines-induced NF-κB p65 nuclear translocation. (**A**) HUVECs were pretreated with or without 10 μmol/L Bay11 for 30 min followed by 10 ng/mL IL-1β or 10 ng/mL TNFα for 24 h. VCAM-1 and ICAM-1 were analyzed by western blotting. Upper panel shows a representative blot (n = 3) and the lower panel the quantitative analysis. *Indicates P < 0.05. (**B**) HUVECs were pretreated with or without 1 μmol/L BI605906 or 100 nmol/L NG25 for 30 min followed by 10 ng/mL IL-1β or 10 ng/mL TNFα for 24 h. VCAM-1 and ICAM-1 were analyzed by western blotting. (**C**,**D**) HUVECs were treated with 10 ng/mL TNFα (**C**) or 10 ng/mL IL-1β (**D**) for indicated time. The nuclear fraction (Nuc) and cytoplasmic fraction (Cyto) were separated. p65 and p50 in each fraction were analyzed by western blotting. (**E**,**F**) HUVECs were pretreated with 5 mmol/L 3-MA, 10 μmol/L MG-132, 10 μmol/L Bay11, or 1 μmol/L BI605906 for 30 min followed by 10 ng/mL TNFα (**E**) or 10 ng/mL IL-1β (**F**) for 6 h. p65 in nuclear fraction was analyzed by western blotting. Upper panel shows a representative blot and the lower panel the quantitative analysis. Each bar denotes mean ± SD of three independent experiments. *Indicates P < 0.05.

Previous reports indicate that TNFα-induced VCAM-1 and ICAM-1 transcription is mediated by NF-κB activation^20^. We confirmed that Bay11, a potent inhibitor of NF-κB activation, blocked VCAM-1 and ICAM-1 expression induced by TNFα or IL-1β (Fig. 5A). We also tested a specific inhibitor of IKKβ, BI605906, and a specific inhibitor of TAK1, NG25, on VCAM-1 and ICAM-1 expression. While BI605906 inhibited both VCAM-1 and ICAM-1 expression induced by TNFα or IL-1β as well as Bay11, NG25 only inhibited TNFα-induced VCAM-1 and ICAM-1expression (Fig. 5B). Since autophagy inhibitors suppressed VCAM-1 transcription only at 6 h, we wondered whether autophagy regulates NF-κB nuclear translocation. We analyzed cytokine-induced nuclear translocation of p65 and p50 proteins in HUVECs at different time points up to 6 h. TNFα increased p65 and p50 in nuclear fraction rapidly at 15 min which persisted for 6 h (Fig. 5C). IL-1β increased p65 and p50 in nuclear fraction with a time-course similar to TNFα (Fig. 5D). To evaluate the effect of 3-MA on p65 nuclear translocation, we compared p65 levels in nuclear fractions of 3-MA treated cells vs. MG-132, Bay11 or BI605906 treated cells. MG-132, Bay11 and BI605906 markedly reduced nuclear p65 at 6 h (Fig. 5E,F). 3-MA also reduced nuclear p65 at 6 h after addition of TNFα (Fig. 5E) or IL-1β (Fig. 5F). This result is consistent with the qPCR data in that 3-MA inhibited VCAM-1 mRNA level at 6 h (Fig. 4B). These results suggest that autophagy facilitates NF-κB nuclear translocation at least up to 6 h after cytokine treatment.

### Autophagy contributes to the late-phase IκBα degradation

**Figure 6:**
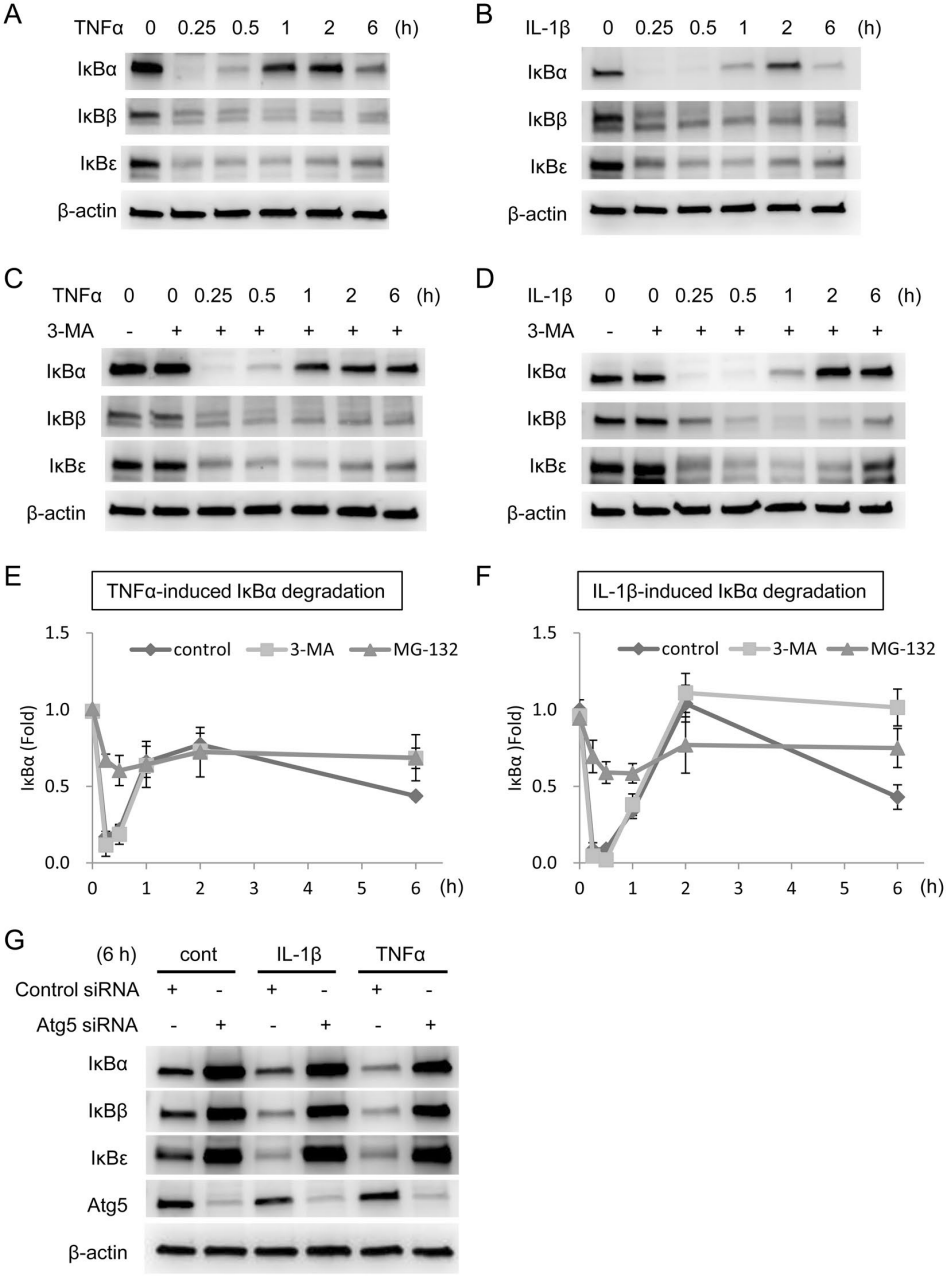
3-MA inhibits late-phase IκBα degradation. (**A**–**D**) HUVECs were pretreated with or without 5 mmol/L 3-MA for 30 min followed by 10 ng/mL TNFα (**A**,**C**) or 10 ng/mL IL-1β (**B**,**D**) for indicated time. IκBα, IκBβ and IκBε were analyzed by western blotting. (**E**,**F**) IκBα blots of TNFα or IL-1β control vs. 3-MA or MG-132 treatment (n = 3) at each time point were quantified by densitometry. (**G**) HUVECs were transfected with siRNA for 72 h followed by 10 ng/mL IL-1β or 10 ng/mL TNFα for 6 h. IκBα, IκBβ, IκBε and Atg5 were analyzed by western blotting.

Since 3-MA reduced the nuclear p65 level at 6 h, we suspected that autophagy may regulate the level of IκB proteins. We carried out experiments to determine cytokine-induced IκBs (IκBα, IκBβ and IκBε) degradation at different time points. As shown in Fig. 6A, IκBα was degraded rapidly and became barely detectable at 15–30 min but was resynthesized and the level was increased at 1 h and 2 h after TNFα treatment. It went through a late-phase degradation and its level became reduced at 6 h (Fig. 6A). IκBβ was also rapidly degraded but was not resynthesized and remained low for 6 h (Fig. 6A). IκBε was rapidly degraded and its level remained low until at 6 h when it started to rise (Fig. 6A). IL-1β induced a biphasic IκBα degradation in a manner closely resembling TNFα except a delay in IκBα resynthesis: its level remained barely detectable at 1 h (Fig. 6B). However, its level at 2 h was restored to the basal level (Fig. 6B). Like TNFα, IL-1β also caused a rapid monophasic degradation of IκBβ and IκBε (Fig. 6B). We next evaluated the effect of 3-MA on degradation of IκBs in HUVECs. 3-MA did not affect TNFα- or IL-1β-induced degradation of IκBα at 15 min or 30 min nor did it influence IκBα resynthesis at 1 and 2 h, but inhibited IκBα degradation at 6 h (Fig. 6C,D). IκBα increase at 6 h by 3-MA is correlated with decrease of nuclear p65 at 6 h (Fig. 5E,F). 3-MA did not affect cytokine-induced IκBβ or IκBε degradation throughout the time-course (Fig. 6C,D). IκBs are well known to be degraded by proteasome. To test whether 3-MA inhibits IκBα degradation by inhibiting proteasomal degradation, we compared the effect of 3-MA with that of MG-132. MG-132 blocked TNFα- or IL-1β-induced IκBα degradation at 15 min and 30 min as well as 6 h and also inhibited IκBβ and IκBε degradation throughout the time-course (Fig. S1). Furthermore, MG-132 inhibited VCAM-1 and ICAM-1 protein expression at 6 h (Fig. S2), which is consistent with the inhibition of IκBα degradation at early time points. As MG-132 caused cell death after incubation with HUVECs for 24 h, we could not determine whether MG-132 influences VCAM-1 or ICAM-1 expression at 24 h. Figure 6E,F show the time-course of TNFα- or IL-1β-induced IκBα degradation in the presence of 3-MA or MG-132 for 6 h. These results suggest that TNFα- and IL-1β-induced biphasic IκBα degradation is controlled by different degradation mechanisms; the early phase degradation is processed by the proteasome machinery while the late phase by autophagy. While 3-MA has no effect on the expression of IκBs at basal state, transfection of Atg5 siRNA dramatically increased the level of IκBs at basal state and enhanced IκBα at 6 h after cytokines stimulation (Fig. 6G). This result suggests that autophagy is involved in not only the late-phase degradation of IκBα, but also the homeostasis of all IκB proteins. Together with the results of nuclear translocation of NF-κB p65 and transcription of VCAM-1, these findings suggest that cytokine-induced autophagy mediates late-phase IκBα degradation, prolongs NF-κB p65 stay in nucleus and facilitates long-term VCAM-1 transcription.

### 3-MA inhibits TNFα- and IL-1β-induced IP-10 (CXCL10) expression

**Figure 7:**
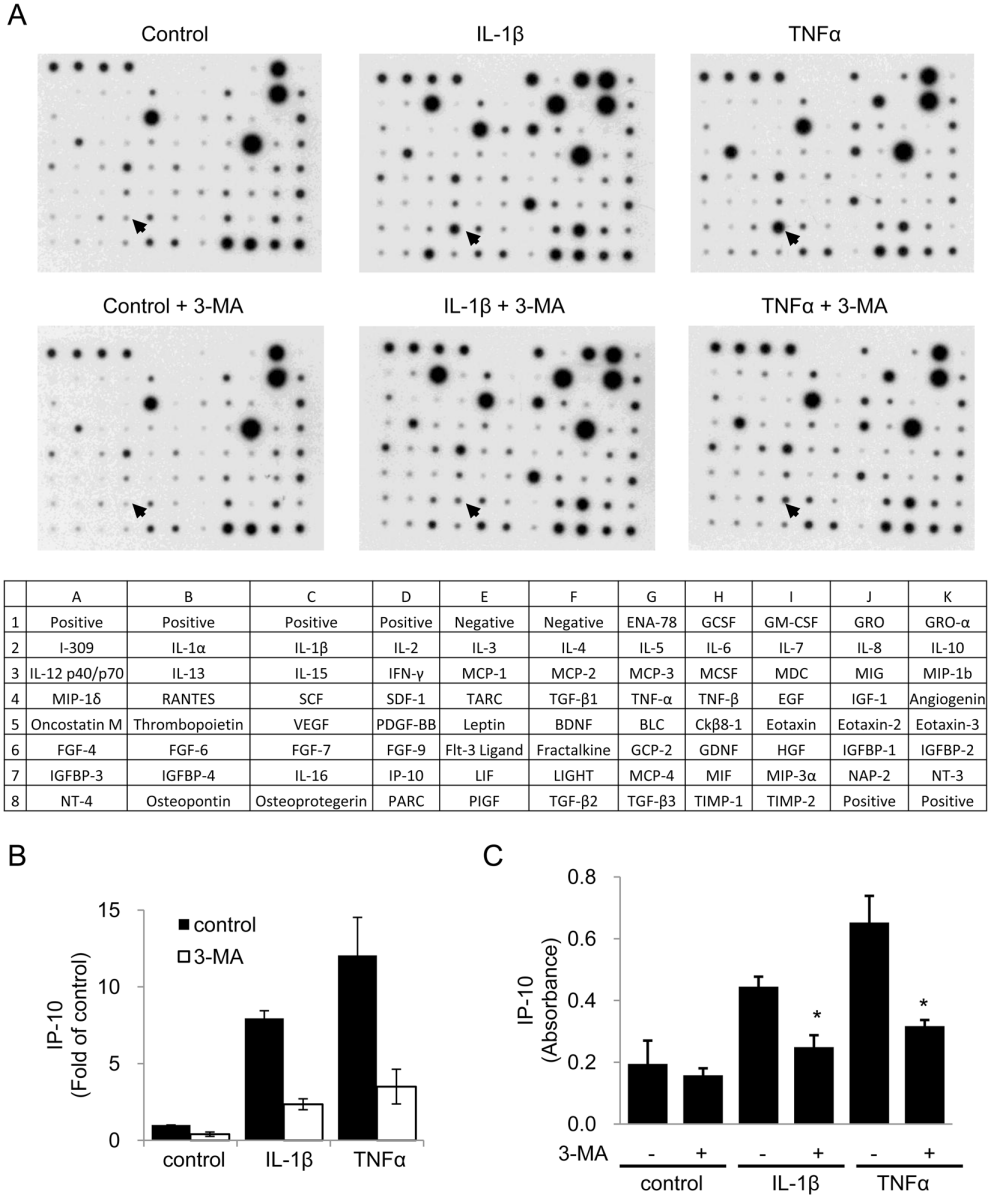
3-MA inhibits cytokines-induced IP-10 expression. (**A**) HUVECs were pretreated with or without 5 mmol/L 3-MA for 30 min followed by 10 ng/mL IL-1β or 10 ng/mL TNFα for 24 h. Conditioned medium was collected and cytokines in the medium were analyzed by an antibody array. Upper panel shows representative dot blots of 80 cytokines/chemokines. Arrows indicate dots of IP-10. The lower panel shows the place of each cytokine within the array. (**B**) Densitometric analysis of IP-10 in the dot blots (n = 2). (**C**) IP-10 in the medium was analyzed by ELISA. Each bar denotes mean ± SD of three independent experiments. *Indicates P < 0.05.

Since our results show that autophagy promotes lymphocyte adhesion to endothelial cells, we suspected that autophagy may enhance chemokine expression. We therefore determined the level of pro-inflammatory cytokines/chemokines in HUVEC conditioned medium using a human cytokine antibody array which targets a panel of 80 cytokines, chemokines and growth factors. At basal state, HUVECs released a large amount of GRO (CXCL1), IL-8 (CXCL8), MCP1 (CCL2) and EGF into the medium (Figs 7A and S3). Compared to the basal state, IL-1β increased the level of 12 cytokines/chemokines, notably ENA78 (CXCL5), GM-CSF, IL-6, Rantes, GCP2 (CXCL6), MIP-3α and IP-10 (CXCL10) (Figs 7A and S3). TNFα increased 8 cytokines/chemokines, notably IL-6, Rantes, IP-10 and MIP-3α (Figs 7A and S3). 3-MA did not alter the basal state of cytokines/chemokines level (Figs 7A and S4). It suppressed 5 of 12 IL-1β-induced cytokines/chemokines including ENA78, MCP2, IP-10, GM-CSF and osteoprotegerin and 2 of 8 TNFα-induced cytokines/chemokines including Rantes and IP-10 (Figs 7A and S4). Among IL-1β- and TNFα-induced cytokines/chemokines, IP-10 was the only common target reduced by 3-MA (Fig. 7B). To provide additional evidence, we analyzed the effect of 3-MA on IL-1β- and TNFα-induced IP-10 by ELISA. 3-MA significantly reduced IL-1β- and TNFα-stimulated IP-10 (Fig. 7C). These results suggest that autophagy also regulates IP-10 expression.

## Discussion

Findings from this study provide new information about regulation of VCAM-1 expression in endothelial cells stimulated by pro-inflammatory cytokines. We show that cytokines-induced autophagy promotes the late-phase degradation of IκBα, prolongs nuclear NF-κB p65 translocation and facilitates long-term VCAM-1 transcription. Our results show for the first time that autophagy activation is essential for sustained VCAM-1 expression in HUVECs stimulated by TNFα or IL-1β.

It was reported that IκB proteins can be degraded in lysosome via a phosphorylation/ubiquitination independent mechanism^21^. After the report of biphasic IκBα degradation following cellular stress signals^22,23^, it was suggested that IκBα degradation is mediated by autophagy in epithelial cells^9^. Our results of IκBα degradation in endothelial cells with 3-MA and MG-132 prove that only the late-phase IκBα degradation is processed by autophagy while early-phase IκBα degradation occurs in the proteasome. Degradation of IκBβ and IκBε is processed by proteasome but not mediated by autophagy. Moreover, we show correlation of decreased NF-κB in nucleus with inhibition of late-phase IκBα degradation, which suggests that resynthesized IκBα is capable of sequestering NF-κB when it is not degraded by autophagy. Although evidence for late-phase IκBα degradation by autophagy is solid, the exact mechanism by which late-phase IκBα degradation is selectively processed by autophagy remains unclear.

VCAM-1 and ICAM-1 share similar structural and functional properties and their expression are promoted by NF-κB activation^5^. It is of interests to note that autophagy-mediated late-phase IκBα degradation regulates long-term VCAM-1 expression but has no effect on sustained ICAM-1 expression. Several reports suggest that VCAM-1 and ICAM-1 can be differentially regulated^5,24^, but the mechanism by which VCAM-1 and ICAM-1 are differentially regulated remains unclear. Our results show for the first time that autophagy is one of the differential regulatory mechanisms for VCAM-1 and ICAM-1 expression. The reason that ICAM-1 expression is not affected by autophagy is not clear. As our data show that degradation of IκBβ and IκBε are not influenced by autophagy, one possible mechanism is that VCAM-1 expression depends on IκBα degradation whereas ICAM-1 expression depends on IκBβ or IκBε. This notion is supported by reported data. It has been shown that IκBε preferentially binds c-Rel^25^. Furthermore, pro-inflammatory mediators-induced ICAM-1 expression was reported to require c-Rel^8^. Further work is needed to resolve the complex mechanisms by which ICAM-1 and VCAM-1 expressions are differentially regulated.

The cytokine array results provide important information about differential regulation of VCAM-1 and ICAM-1. Although expression of most cytokine/chemokine genes stimulated by TNFα or IL-1β in our array was reported to depend on NF-κB transactivation, it is unexpected that IP-10 (Interferon γ-induced protein 10) is the only common target regulated by autophagy. As illustrated in Fig. S5, comparison of the binding motifs at the proximal promoter regions of autophagy-regulated genes (VCAM-1 and IP-10) vs. autophagy-independent genes (ICAM-1, IL-6, GCP2 and MIP-3α) reveals that the proximal promoter regions of VCAM-1 and IP-10 contains two NF-κB binding sites whereas the promoter region of ICAM-1, IL-6, GCP-2 and MIP-3α contains one NF-κB binding site and also C/EBPβ binding motifs^8,26–29^. Alternative possible explanations for the differential effects of autophagy are that autophagy-regulated genes are more sensitive to NF-κB level in nucleus because there are two NF-κB binding sites at the promoter regions and genes not regulated by autophagy are concurrent activated with other trans-activators such as C/EBPβ. These explanations are supported by reported data that promoter activities of VCAM-1 and ICAM-1 exhibited different sensitivity to overexpressed IκBα^25^ and both NF-κB and C/EBPβ binding sites at the promoter region are required for ICAM-1 expression^30^. The underlying mechanisms are likely to be more complex and need to be further studied.

IP-10 (CXCL10) is a CXC motif chemokine produced by several cell types including endothelial cells, monocytes and fibroblasts^31^. It attracts monocytes and T lymphocytes and is reported to play an important role in promoting T lymphocytes adhesion to endothelial cells^32,33^. It is interesting that autophagy concurrently upregulates IP-10 and VCAM-1 as VCAM-1 is a ligand for lymphocyte VLA4 (integrin α_4_β_1_) and is a key adhesion molecule on endothelial cells for lymphocytes adhesion^34^. VCAM-1 was reported to be critical for early atherosclerosis as genetic deletion of VCAM-1 greatly attenuates atherosclerosis in mice^35^. Lymphocytes infiltration is considered to trigger immune reactions in vascular wall during endothelial injury and contributes to progression of atherosclerosis^36,37^. Pro-inflammatory cytokine-induced autophagy may thus play a critical role in enhancing lymphocytes and monocytes adhesion to and transmigration through endothelium in the development of atherosclerosis.

Several reports have shown that pro-inflammatory mediators-induced VCAM-1 and ICAM-1 expression are mediated via p38 MAPK^38,39^. On the other hand, inhibition of ERK with PD98059 or JNK with SP600125 was reported to enhance VCAM-1 expression^40^ suggesting a negative regulation of VCAM-1 by ERK and JNK. Our results are consistent with the reported data but provide additional information regarding the requirement of p38 MAPK activation for short-term (6 h) and long-term (24 h) expression of VCAM-1. Although there are reports about regulation of autophagy by p38 MAPK activation^41,42^, our data do not show such signaling relationship. Furthermore, our results show differential effect of 3-MA and SB202190 on the time-dependent expression of VCAM-1: 3-MA blocks VCAM-1 expression at 24 h without an effect at 6 h whereas SB202190 inhibits VCAM-1 expression at both time points. Taken together, our findings indicate that TNFα- and IL-1β-induced autophagy-dependent VCAM-1 expression in endothelial cells is not mediated by p38 MAPK.

In summary, our findings provide new information regarding the role of autophagy in sustained expression of VCAM-1 and IP-10 in endothelial cells stimulated by pro-inflammatory cytokines. Our results indicate that autophagy enhances VCAM-1 transcription by degrading late-phase IκBα. Persistent expression of VCAM-1 and IP-10 facilitate lymphocyte recruitment and adhesion to endothelium.

## Materials and Methods

### Materials

Recombinant human IL-1β (#8900) and TNFα (#8902) were purchased from Cell Signaling Technology. Rabbit antibodies against LC3B (#3868), Beclin-1 (#3495), phospho-S6K (#9234), S6K (#2708), ICAM-1 (#4915), VCAM-1 (#13662), phospho-p38 (#4511), p38 (#8690), phospho-ERK1/2 (#9101), ERK1/2 (#9102), phospho-JNK (#4668), JNK (#9252), p65 (#8242), p50 (#3035), histone H3 (#9715), IκBα (#9242), and mouse antibodies against IκBβ (#8635) were purchased from Cell Signaling Technology. Rabbit antibodies against IκBε (ab134143) and IP-10 (ab9807) were purchased from Abcam. Rapamycin (R0395), 3-MA (M9281), Bafilomycin A (B1793), Bay11 (B5556), MG-132 (SML1135), SB202190 (S7067), PD98059 (P215), SP600125 (S5567) and NG25 (SML1332) were purchased from Sigma-Aldrich. BI605906 (#19184) was purchased from Cayman. Horseradish peroxidase-conjugated anti-mouse (sc-2005) and anti-rabbit (sc-2004) IgG were purchased from Santa Cruz.

### Cells

Primary HUVECs at passage 2 were purchased from Bioresource Collection and Research Center (Hsinchu, Taiwan) and maintained in HUVEC growth medium (EBM-2, Lonza) with full supplements (2% fetal bovine serum, 0.4% human fibroblast growth factor-2, 0.1% vascular endothelial growth factor, 0.1% R^3^-insulin-like growth factor-1, 0.1% human epidermal growth factor, 0.04% hydrocortisone, 0.1% ascorbic acid, 0.1% GA-1000). Only passages 3 to 6 cells were used. Cells were treated with chemical inhibitors for 30 min before addition of inflammatory cytokine. At the indicated time point, cells were lysed with ice-cold lysis buffer (20 mmol/L Tris-HCl, pH 7.5, 150 mmol/L NaCl, 1 mmol/L EDTA, 1 mmol/L EGTA, 1% CHAPS, 2.5 mmol/L sodium pyrophosphate, 1 mmol/L β-glycerophosphate, 1 mmol/L Na3VO_4_) and protease/phosphatase inhibitors cocktail (Thermo Scientific) immediately.

### Immunoblotting

Cells were scraped and lysed in lysis buffer on ice for 5 min. Lysates were then centrifuged at 12,000 g for 5 min to remove cell debris. Supernatants containing 25 μg of protein were mixed with Laemmli sample buffer (Bio-Rad) and boiled for 5 min before being subjected to electrophoresis in 4-15% sodium dodecyl sulfate-polyacrylamide gels and then transferred to polyvinylidene difluoride membranes (Millipore). For detection, membranes were blocked with 5% bovine serum albumin in 25 mmol/L Tris-HCl, pH 7.4, 150 mmol/L NaCl, and 0.05% Tween 20 for 1 h at 22 °C and then incubated with 1:1000 diluted primary antibodies overnight at 4 °C. Membranes were incubated with 1:3000 diluted horseradish peroxidase-linked secondary antibodies for 1 h at 22 °C and then developed with enhanced chemiluminescence substrate (Thermo Scientific). The blots were exposed with a luminescent image analyzer (ImageQuant LAS 4000, GE Healthcare) and quantified by ImageJ software (National Institutes of Health).

### Lymphocyte adhesion assay

Adhesion of Jurkat cells on HUVECs was tested with Leukocyte-Endothelium Adhesion Assay kit (Cell Biolabs) following the manufacturer’s instruction. In brief, HUVECs (50,000/well) were cultured on 0.1% gelatin coated 96-well plate for 48 h and then treated with cytokine with or without inhibitors for 24 h. Leukotracker-labeled Jurkat cells (20,000/well) were then added onto HUVECs for 1 h. After adhesion, cells were lysed and adhesion was determined by the fluorescence at 480 nm/520 nm in the lysates with a Synergy H1 Hybrid Reader (BioTek).

### siRNA transfection

Atg5 siRNA (#6345) was purchased from Cell Signaling Technology. Control siRNA (AM4611) was purchased from Thermo Fisher Scientific. siRNA was transfected into cells with Lipofectamine RNAiMAX kit (Thermo Fisher Scientific) following manufacturer’s instructions. In brief, HUVECs were grown in 0.1% gelatin coated 6-well plate to 80% confluence. siRNA was diluted in Opti-MEM medium and mixed with Lipofectamine RNAiMAX reagent for 5 min at room temperature before transfection. Cells were transfected with the mixture containing 25 pmol siRNA per well and incubated for 72 h.

### Real-time PCR

mRNA was purified from cell lysates with Direct-zol RNA MiniPrep kit (Zymo research). First-strand cDNA was synthesized by incubating 1 μg RNA and SuperScript III First-Strand Synthesis SuperMix (Invitrogen) for 30 min at 50 °C. Real-time quantitative PCR was performed using standard protocols on a CFX96 Real-time System (Bio-Rad) equipped with a 96-well reaction plate. In brief, 40 ng cDNA was mixed with FastStart SYBR Green Master (Roche), 300 nM of each primer and DEPC water to 25 µl. PCR reaction was amplified in duplicate for 15 sec at 95 °C and 1 min at 60 °C for 40 cycles. The primer sequences for each gene are shown in Fig. S6.

### Cell Fractionation

Cells were pretreated with or without chemical inhibitors for 30 min and then incubated with cytokines for indicated time. Cells were fractioned with NE-PER Nuclear and Cytoplasmic Extraction kit (Thermo Fisher Scientific) following manufacturer’s instructions. In brief, cells were lysed with CER I and CER II buffer for 5 min on ice. The cytoplasmic fractions (supernatant) were collected by centrifugation at 16,000 g for 5 min. The nuclear fractions were extracted from the pellet with NER buffer and then collected by centrifugation at 16,000 g for 10 min.

### Cytokine array and ELISA

Cells were pretreated with or without 3-MA for 30 min and then incubated with cytokines for another 24 h. The conditioned medium (CM) was collected and centrifuged at 1,500 g for 5 min to remove cell debris. The supernatant was collected and stored at −20 °C for no more than 1 week. For cytokine array, CM was analyzed with Cytokine Human Membrane Antibody Array Kit (Abcam) following the manufacturer’s instruction. In brief, the array membranes were blocked by blocking buffer for 30 min and then incubated with CM overnight at 4 °C. After incubation with CM, membranes were incubated with biotin-conjugated anti-cytokines overnight at 4 °C and then with HRP-Conjugated Streptavidin for 2 h. The signal was detected with Detection Buffer and exposed on X-ray films (Kodak). For ELISA, CM was coated on a 96-well plate in 0.1 mol/L NaHCO_3_ at 4 °C overnight. The coated plate was blocked with 5% BSA and then incubated with rabbit polyclonal anti-IP10 (Abcam) at 4 °C overnight. After incubation with the secondary antibody (1:500), tetramethylbenzidine (Thermo Scientific) was added for the color reaction. The reaction was stopped with 0.18 mmol/L H_2_SO_4_ and the absorbance was analyzed at 450 nm with a Synergy H1 Hybrid Reader (BioTek).

### Statistical Analysis

Values were expressed as mean ± SD. Differences between groups were analyzed using One Way ANOVA with SigmaStat software (Systat Software, Inc.). *P* < 0.05 was considered statistically significant.

## Electronic supplementary material

Supplemental information
