# Supplementary material for: Cytokine-induced autophagy promotes long-term VCAM-1 but not ICAM-1 expression by degrading late-phase IκBα
Source: Sci Rep. 2017 Sep 29;7:12472. doi: 10.1038/s41598-017-12641-8 (PMC5622139; doi:10.1038/s41598-017-12641-8)
Supplement: Supplementary file 1 — Supplemental information [file 41598_2017_12641_MOESM1_ESM.pdf]

Cytokine-induced autophagy promotes long-term VCAM-1 but not ICAM-1 expression by degrading late-phase IκBα

Ling-Yun Chu,[1-2] Ying-Chang Hsueh,[3] Hsiao-Ling Cheng,[1-2] and Kenneth K. Wu[1-4*]

[1]Metabolomic Medicine Research Center, China Medical University Hospital, Taichung, Taiwan; [2]Graduate Institute of Biomedical Sciences, China Medical University, Taichung, Taiwan; [3]Institute of Cellular and System Medicine, National Health Research Institutes, Zhunan, Taiwan; and [4]Department of Medical Sciences, National Tsing-Hua University, Hsinchu, Taiwan

* Correspondence to Kenneth K Wu, kkgo@nhri.org.tw

Supplemental Figures:

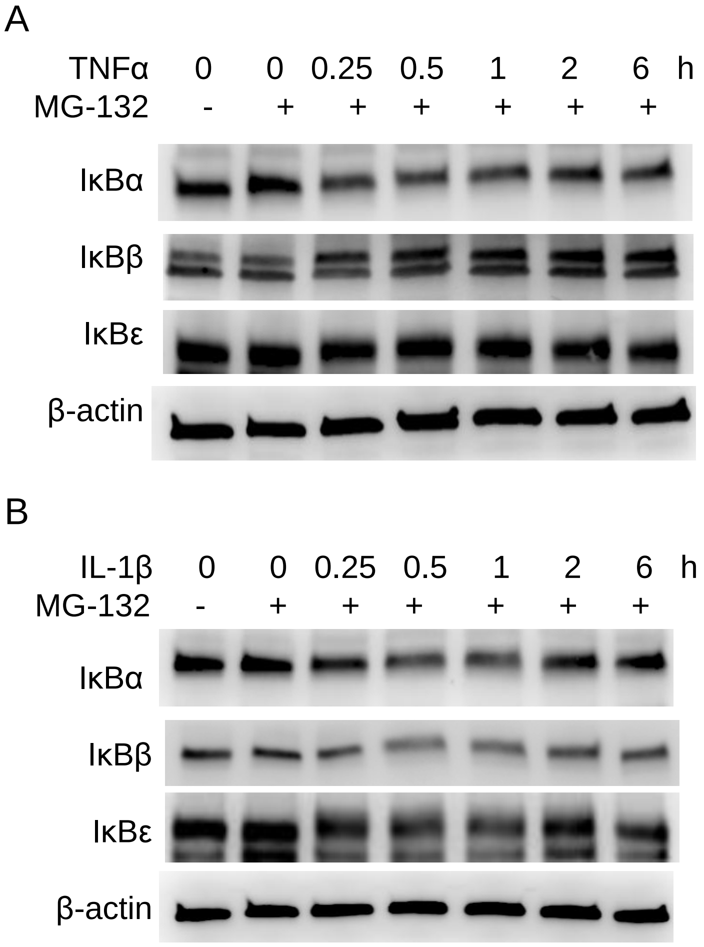

Figure S1. MG-132 inhibits cytokine-induced early-phase degradation of IκBα in HUVECs. (A) HUVECs were pretreated with 10 μmol/L MG-132 for 30 min followed by 10 ng/mL TNFα for indicated time. IκB proteins were analyzed by western blotting. A representative blot of three independent experiments is shown. (B) HUVECs were pretreated with 10 μmol/L MG-132 for 30 min followed by 10 ng/mL IL-1β for indicated time. IκB proteins were analyzed by western blotting. A representative blot of three independent experiments is shown.

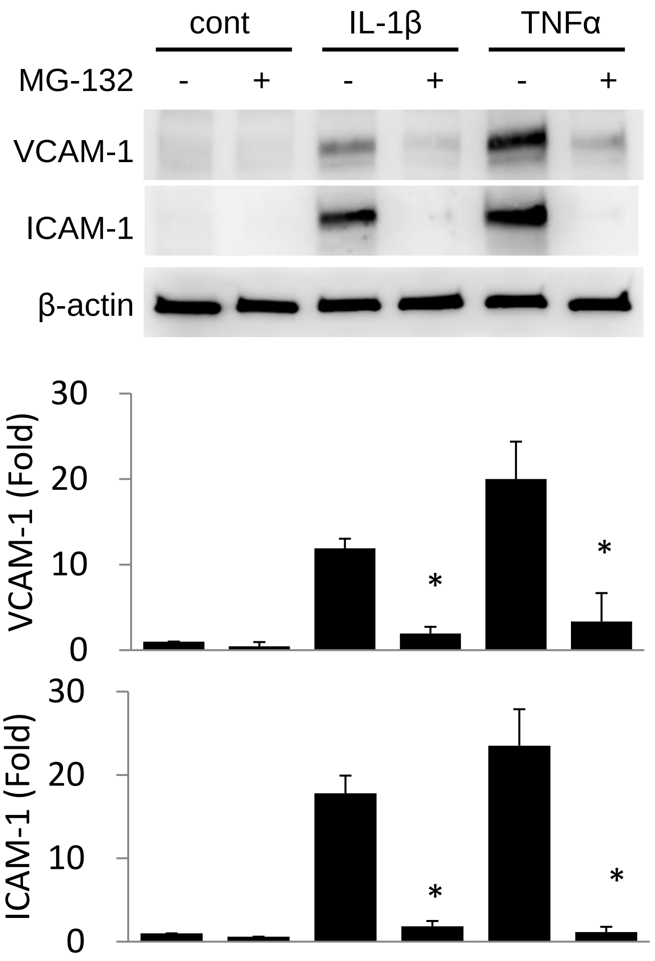

Figure S2. MG-132 inhibits cytokines-induced VCAM-1 and ICAM-1 expression in HUVECs. HUVECs were pretreated with or without 10 μmol/L MG-132 for 30 min followed by 10 ng/mL IL-1β or 10 ng/mL TNFα for 6 h. VCAM-1 and ICAM-1 were analyzed by western blotting. Upper panel shows a representative blot (n=3) and the lower panel the quantitative analysis. * indicates P < .05.

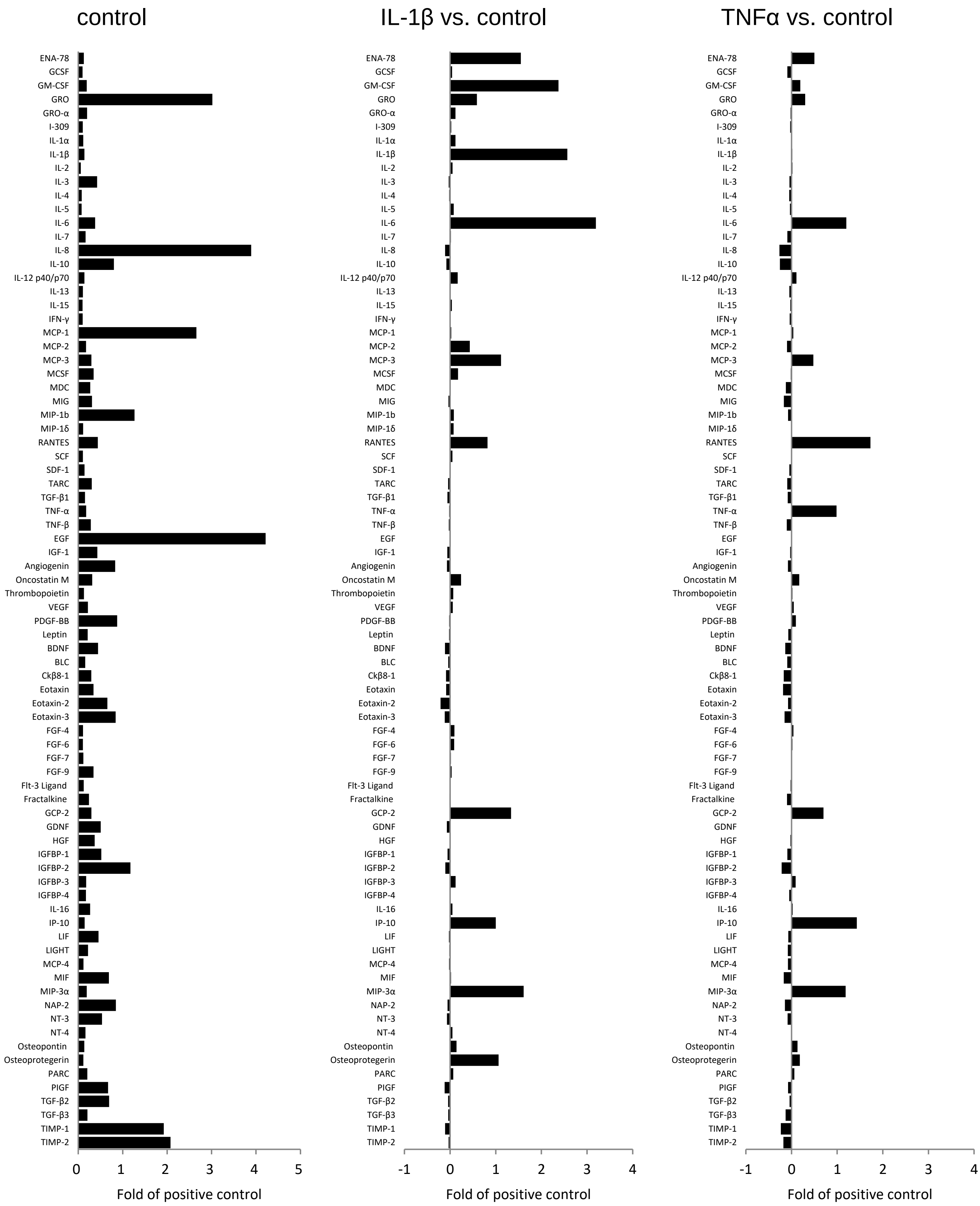

Figure S3. Densitometry analysis of each cytokine/chemokine dot blot as determined by antibody array. HUVECs were treated with 10 ng/mL IL-1β or 10 ng/mL TNFα for 24 h. Cytokines released into medium were analyzed by antibody array. Each dot blot was quantified by densitometry. Each horizontal bar shows increase or decrease compared to control.

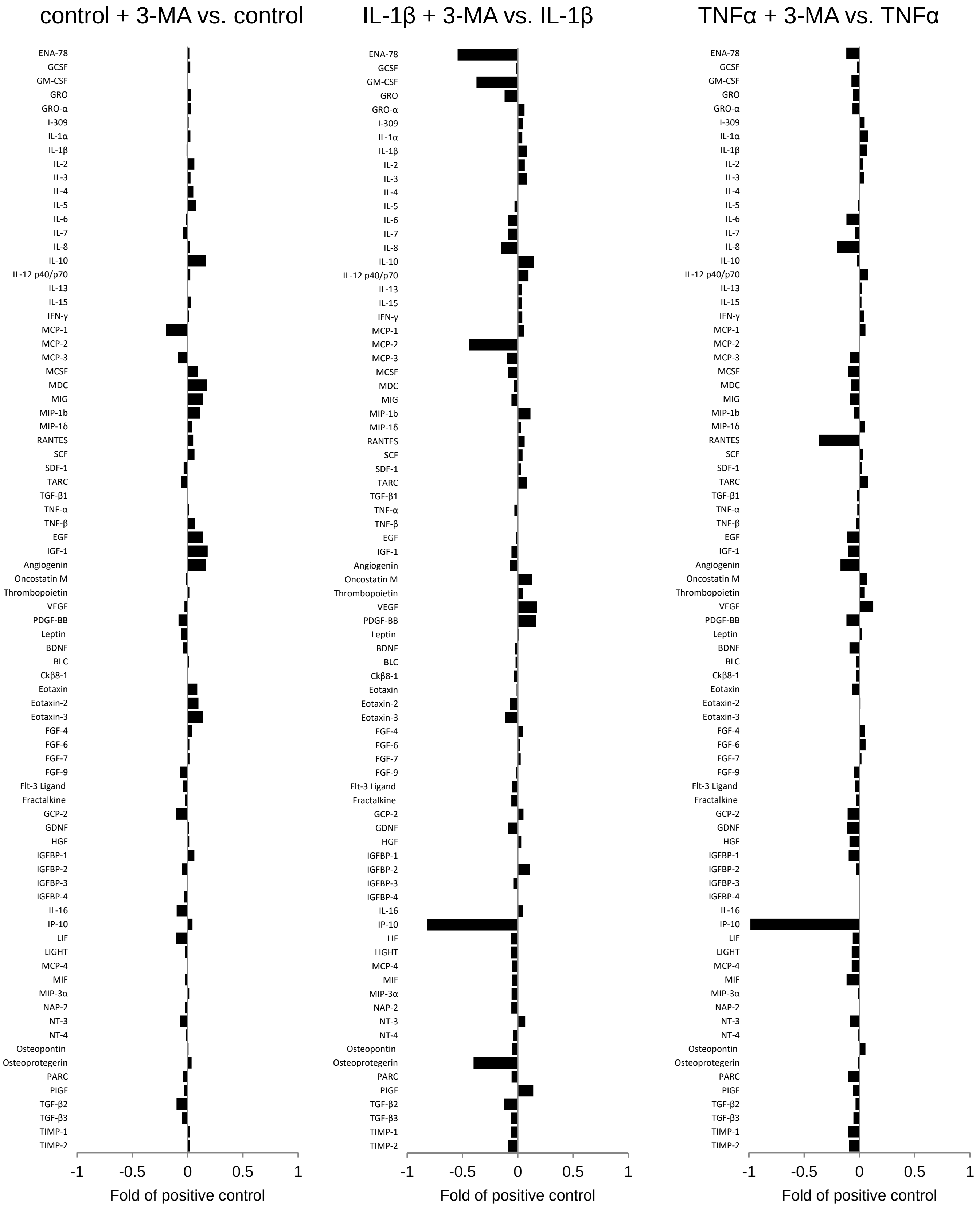

Figure S4. 3-MA reduces IL-1β- and TNFα-induced IP-10 and other cytokines. HUVECs were pretreated with 5 mmol/L 3-MA for 30 min followed by 10 ng/mL IL-1β or 10 ng/mL TNFα for 24 h. Cytokines released into medium were analyzed by antibody array. Each dot blot was quantified by densitometry. Each horizontal bar shows increase or decrease compared to that without 3-MA.

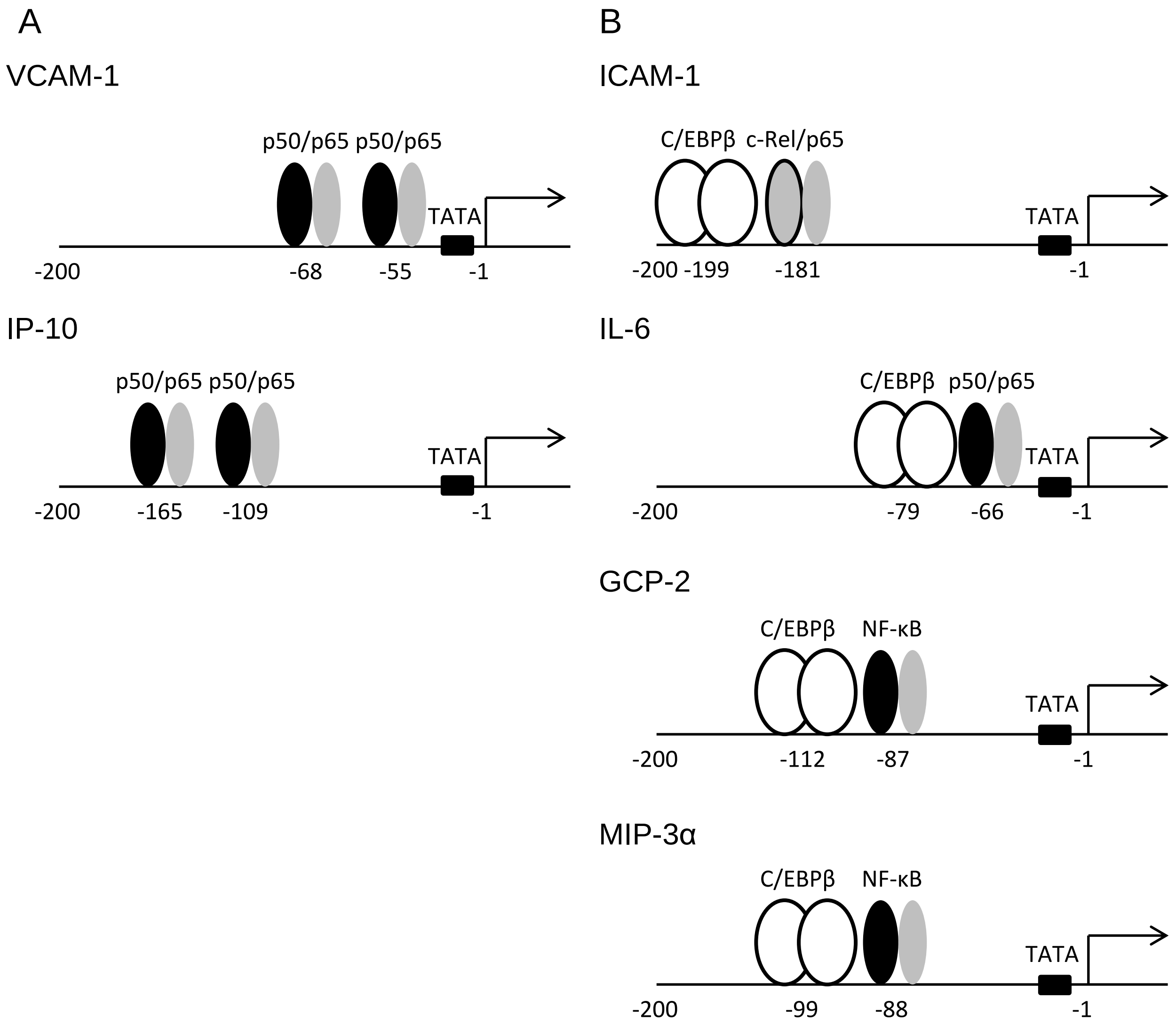

Figure S5. Schematic illustration of the binding motifs in two groups of genes. (A) VCAM-1 and IP-10 promoter regions harbor two NF-κB (p65/p50) binding motifs. This group of genes are regulated by autophagy. (B) ICAM1, IL-6, GCP-2 and MIP-3α promoter regions harbor one NF-κB and one C/EBPβ binding motifs. This group of genes are independent of autophagy regulation.

Gene	species	Forward (5’ → 3’)	Reverse (5’ → 3’)	Product size
cyclophilin A (PPIA)	human	CTCGAATAAGTTTGACTTGTGTTT	CTAGGCATGGGAGGGAACA	165
ICAM-1	human	GGCCGGCCAGCTTATACAC	TAGACACTTGAGCTCGGGCA	166
VCAM-1	human	TCAGATTGGAGACTCAGTCATGT	ACTCCTCACCTTCCCGCTC	109

Figure S6. Sequence of primers for real-time PCR.

Full length blots used in the main figures

Figure 1. A and B

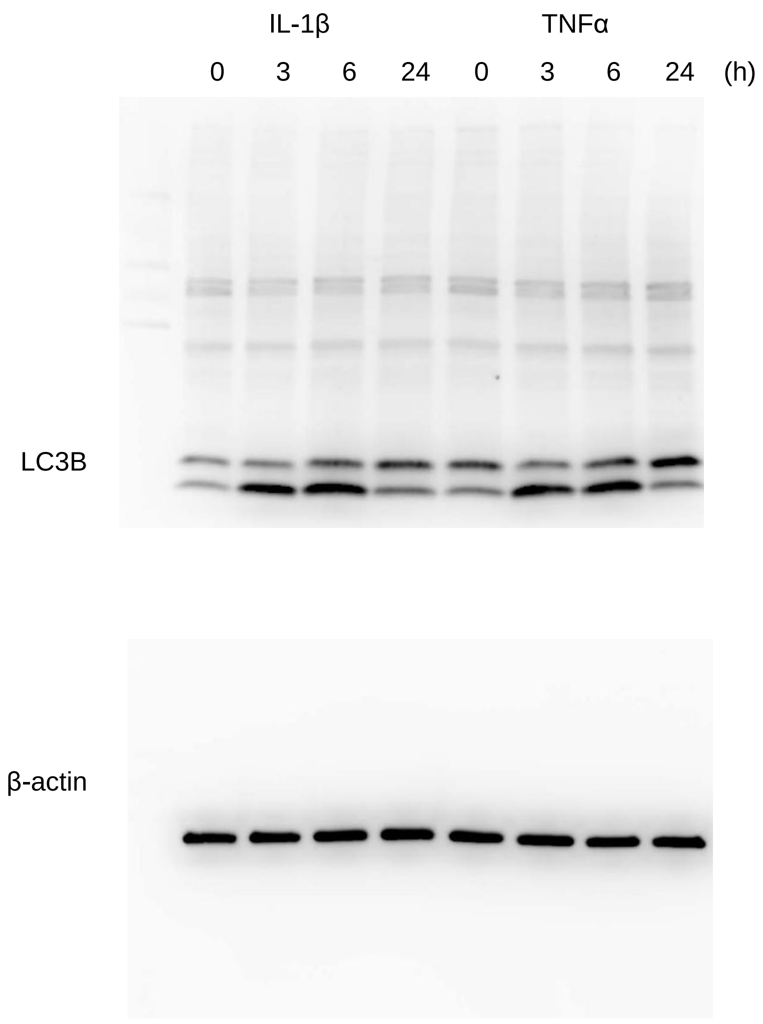

Figure 1. C and D

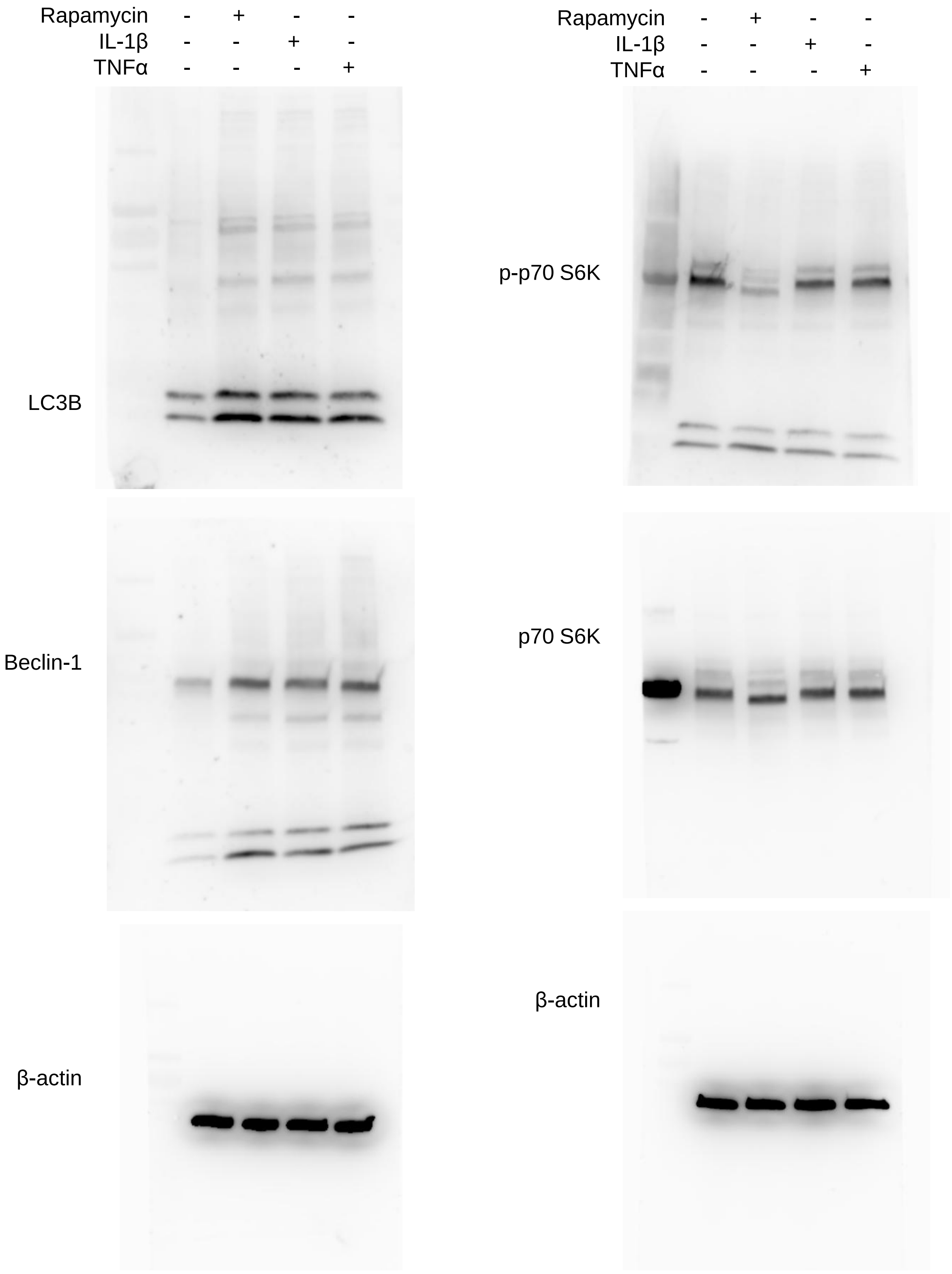

Figure 2. A and B

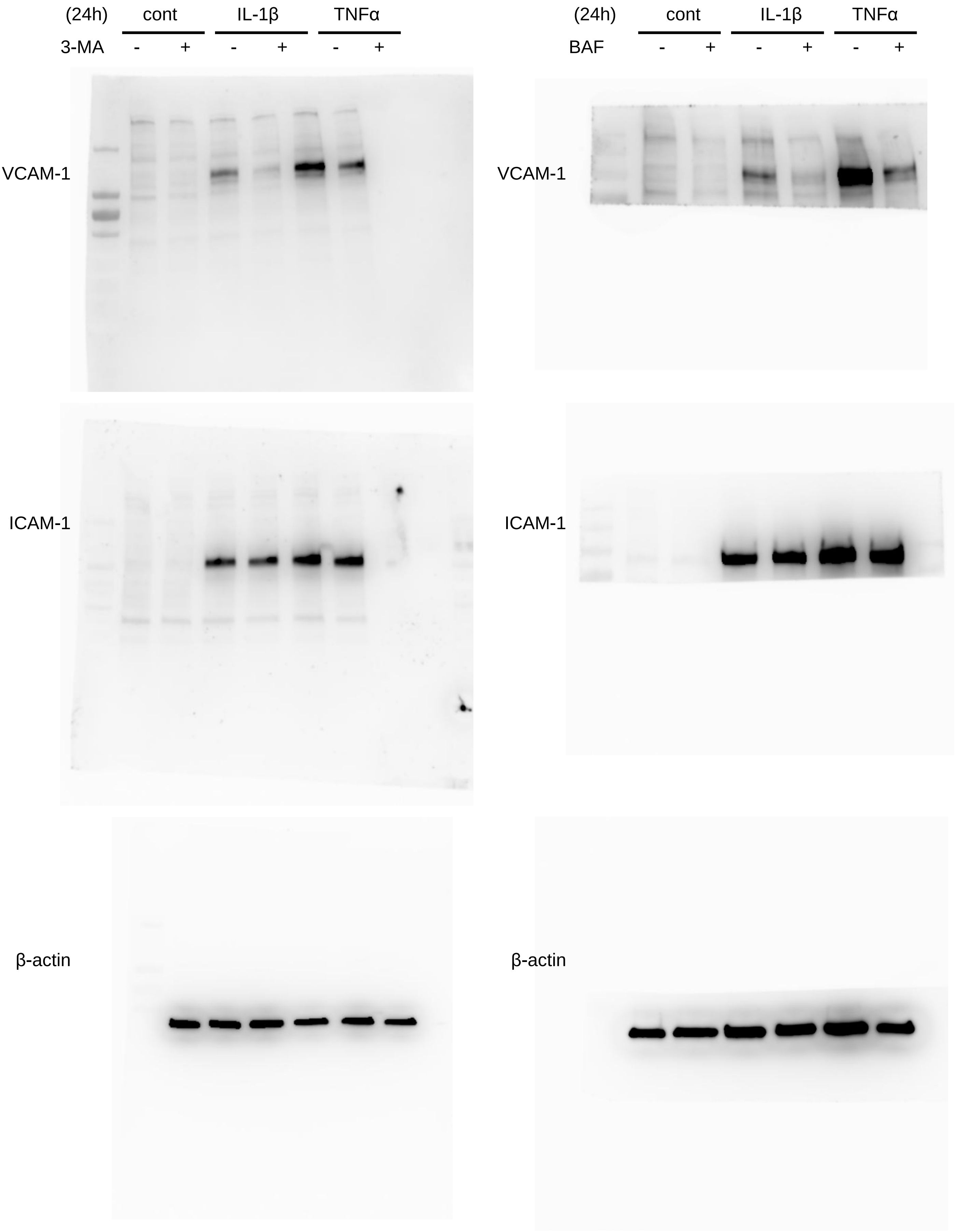

Figure 2. C and D

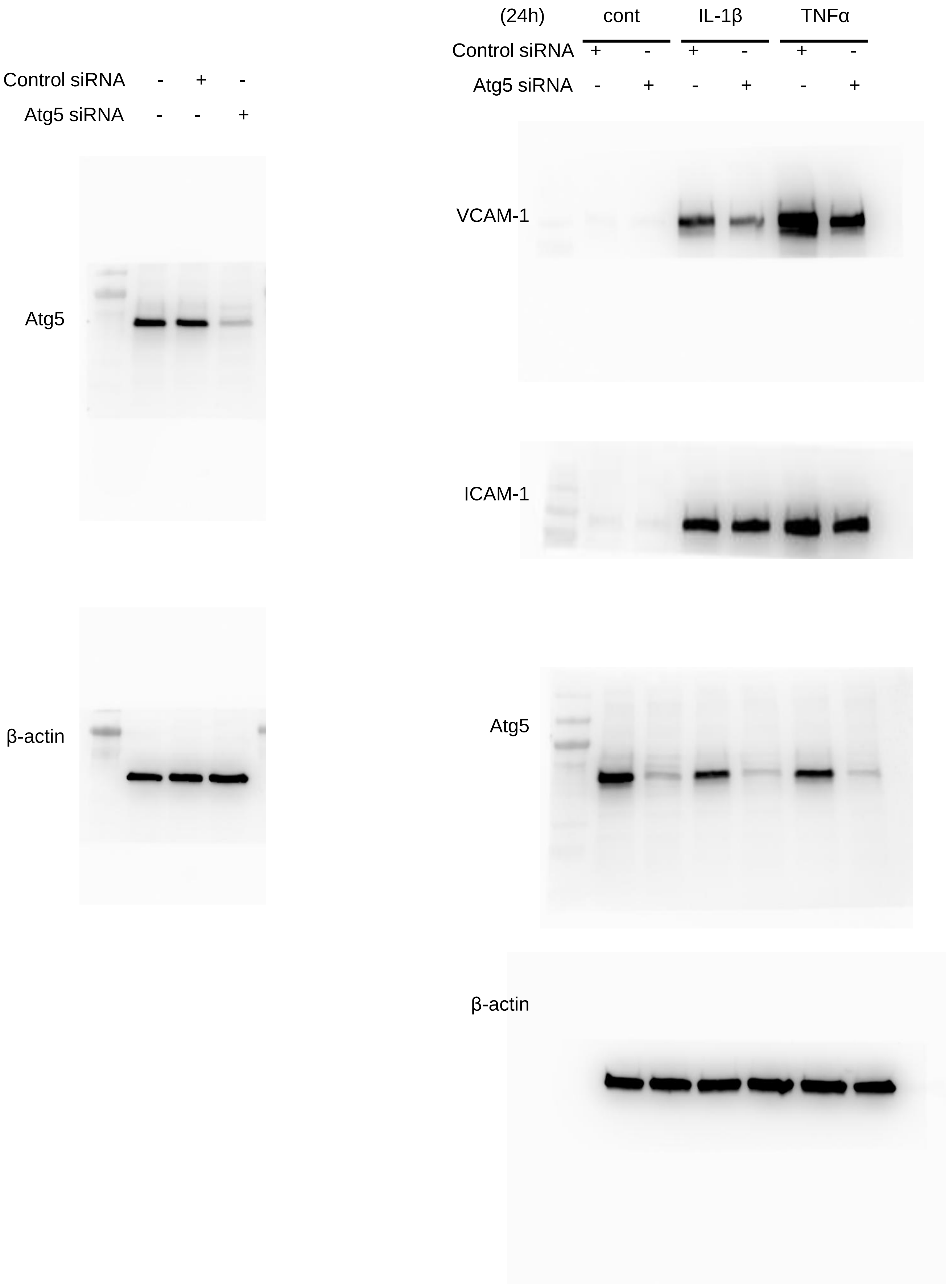

Figure 2. E

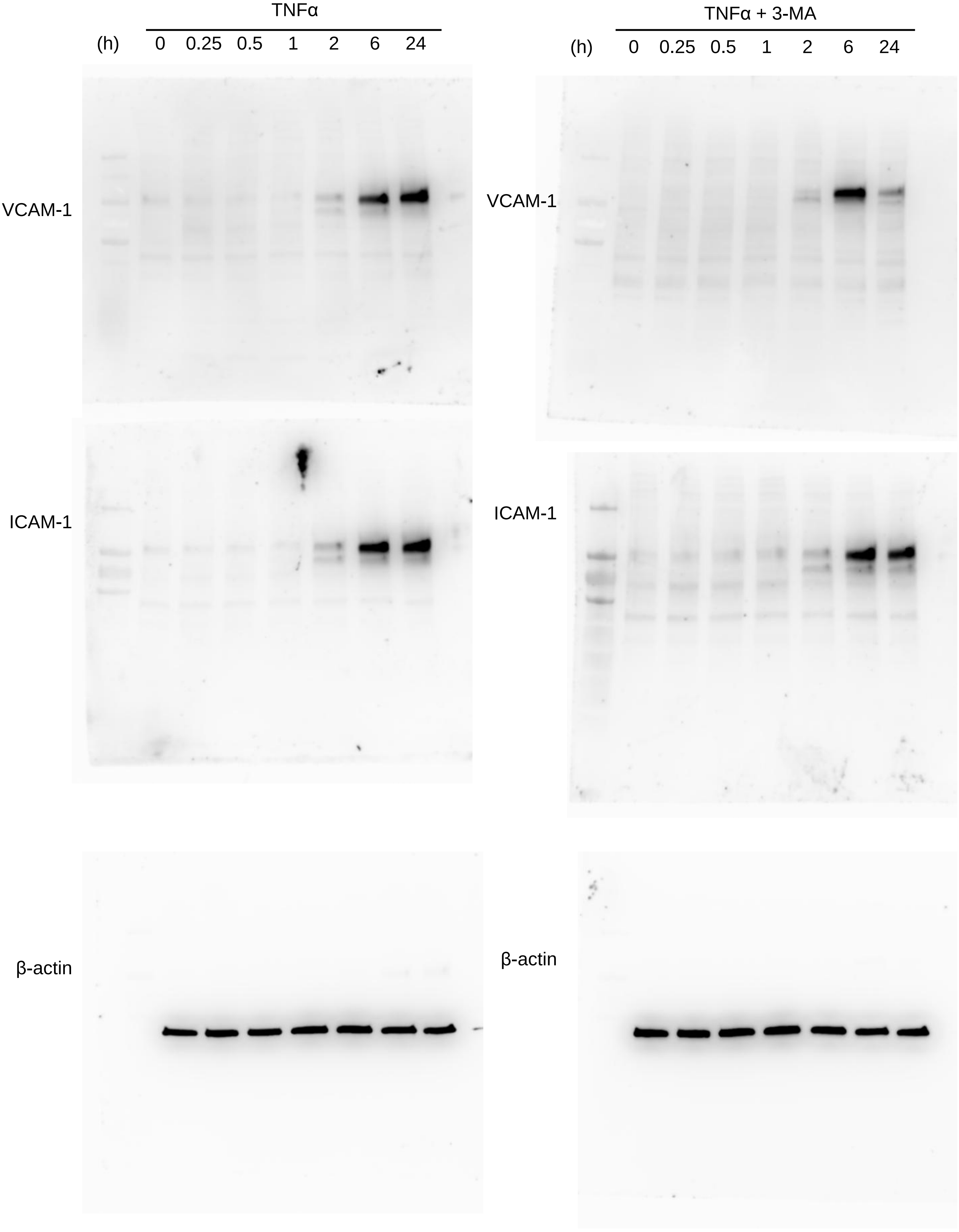
TNFα
(h) 0 0.25 0.5 1 2 6 24
TNFα + 3-MA
(h) 0 0.25 0.5 1 2 6 24
VCAM-1
VCAM-1
ICAM-1
ICAM-1
β-actin
β-actin

Figure 2. F

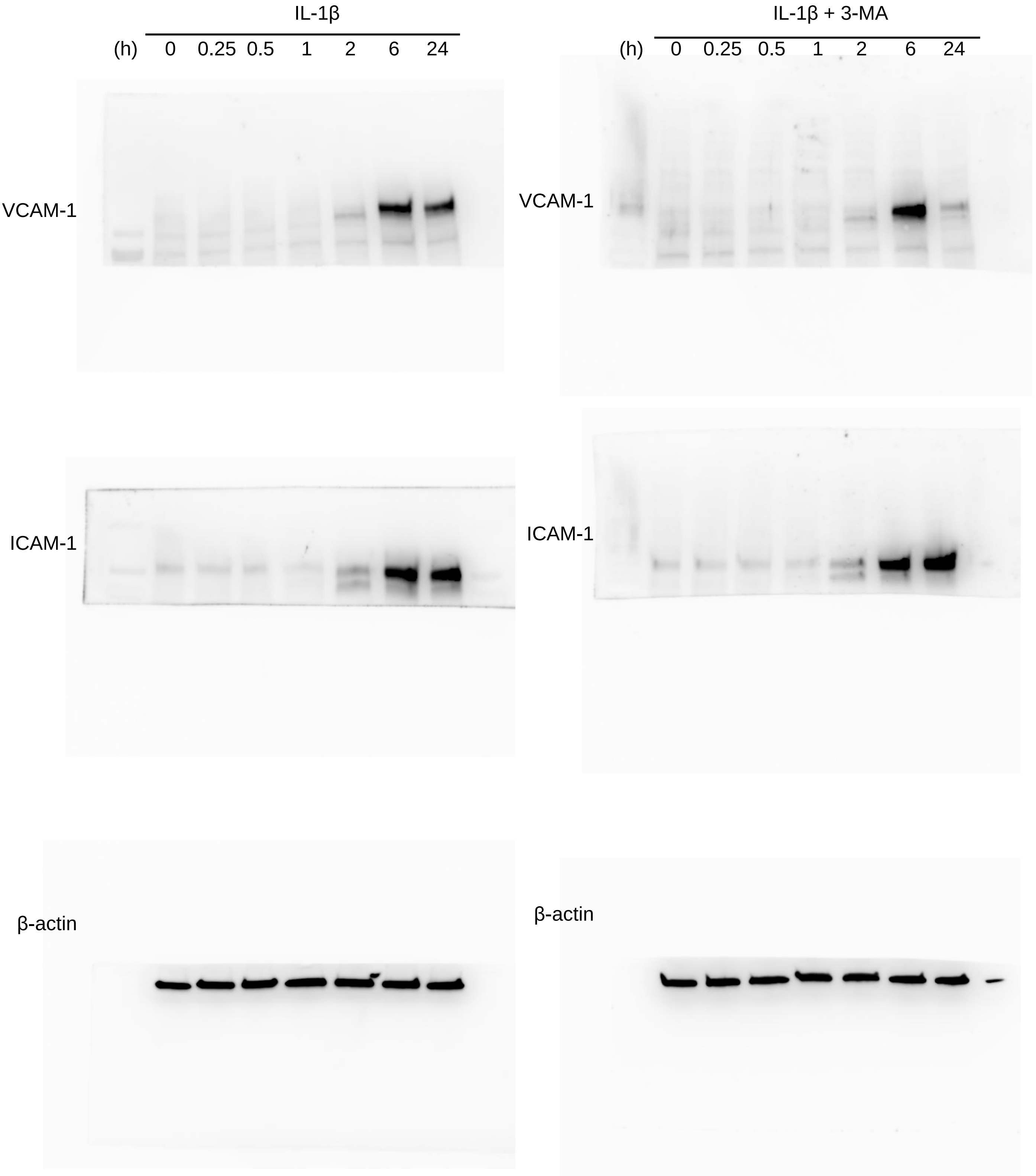
IL-1β
(h) 0 0.25 0.5 1 2 6 24
VCAM-1
ICAM-1
β-actin
IL-1β + 3-MA
(h) 0 0.25 0.5 1 2 6 24
VCAM-1
ICAM-1
β-actin

Figure 3. A

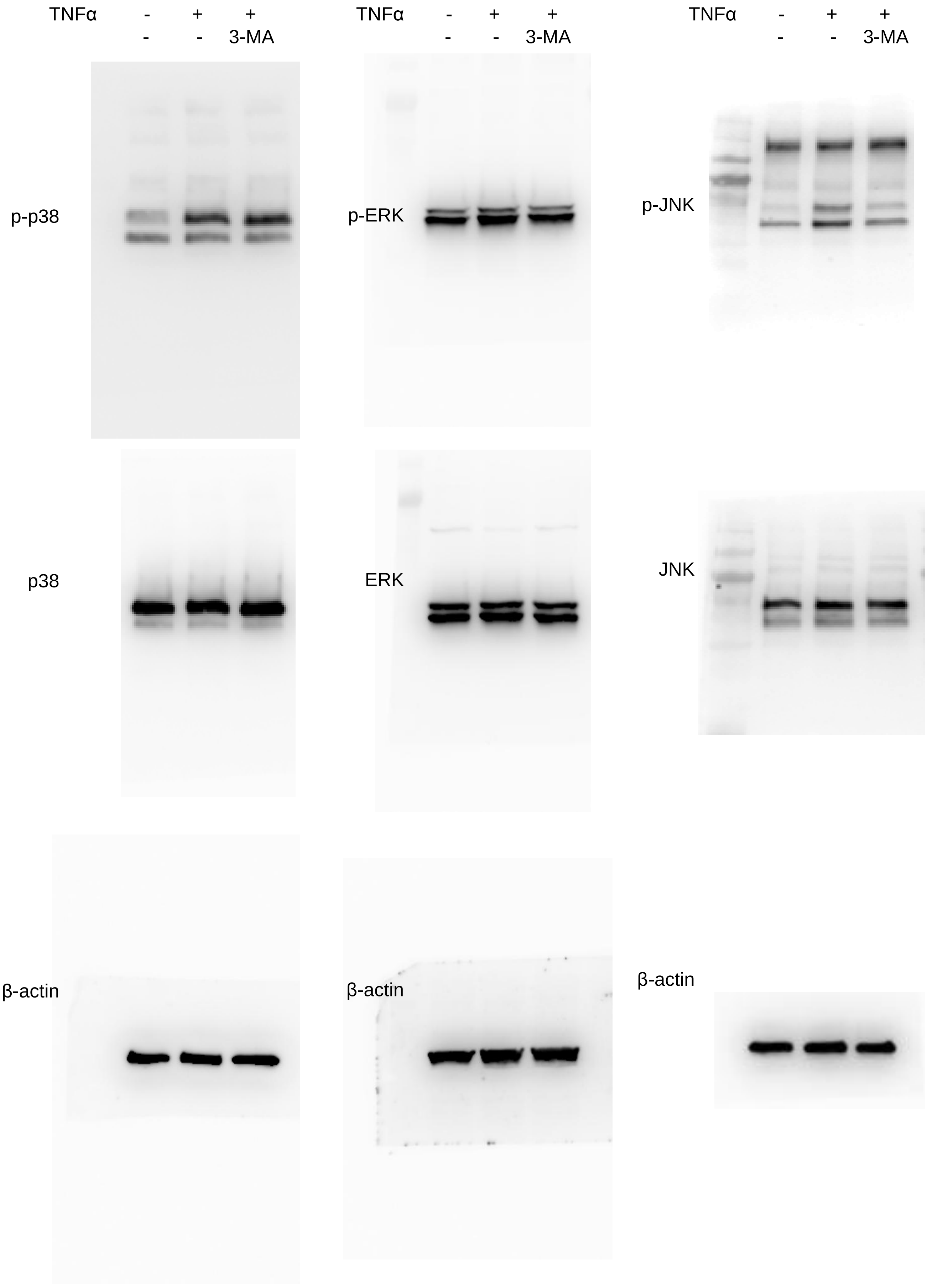
TNFα - + +
- - 3-MA
p-p38
p38
β-actin
TNFα - + +
- - 3-MA
p-ERK
ERK
β-actin
TNFα - + +
- - 3-MA
p-JNK
JNK
β-actin

Figure 3. B

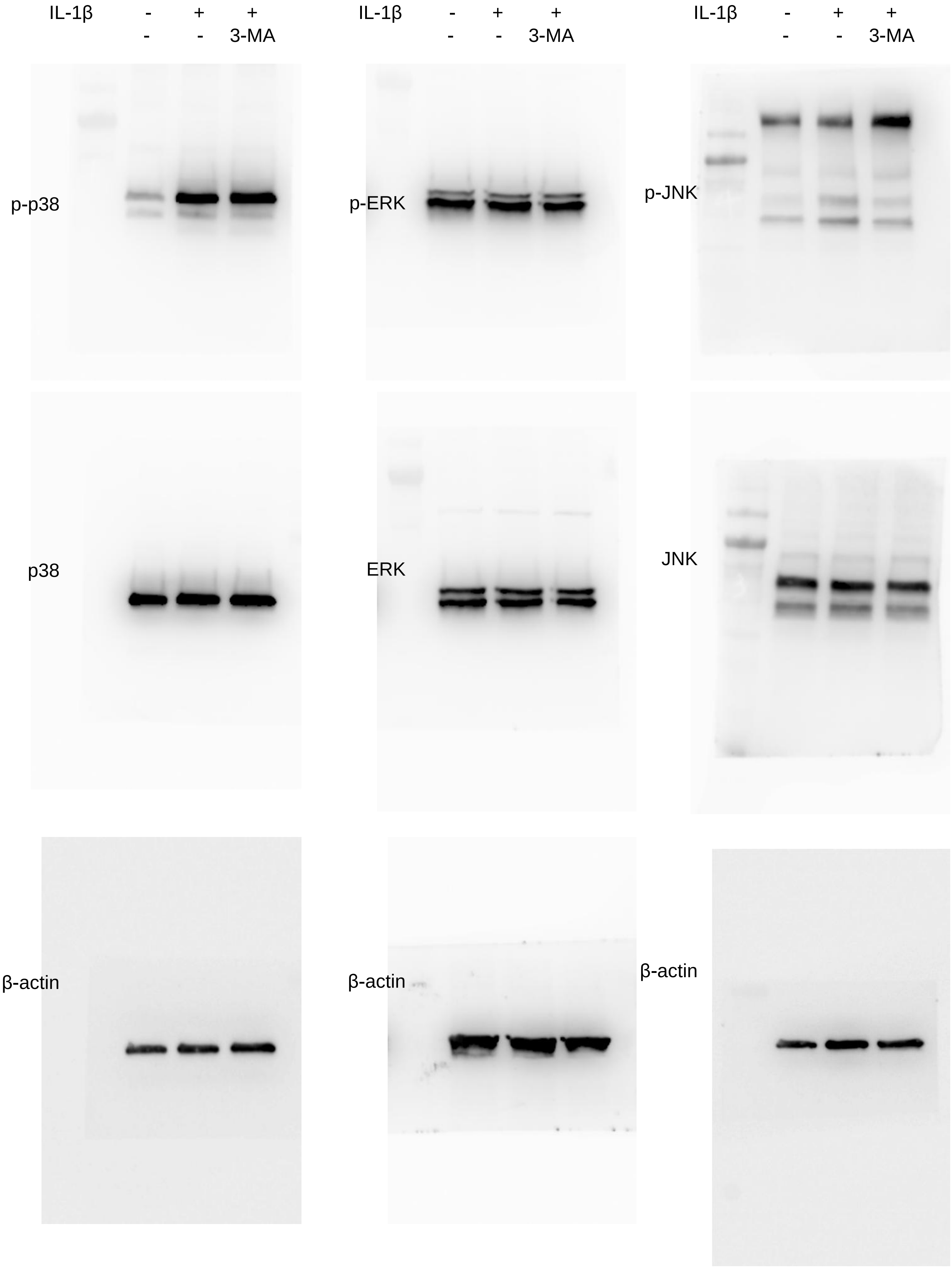
IL-1β - + +
- - 3-MA
IL-1β - + +
- - 3-MA
IL-1β - + +
- - 3-MA
p-p38
p-ERK
p-JNK
p38
ERK
JNK
β-actin
β-actin
β-actin

Figure 3. C and D

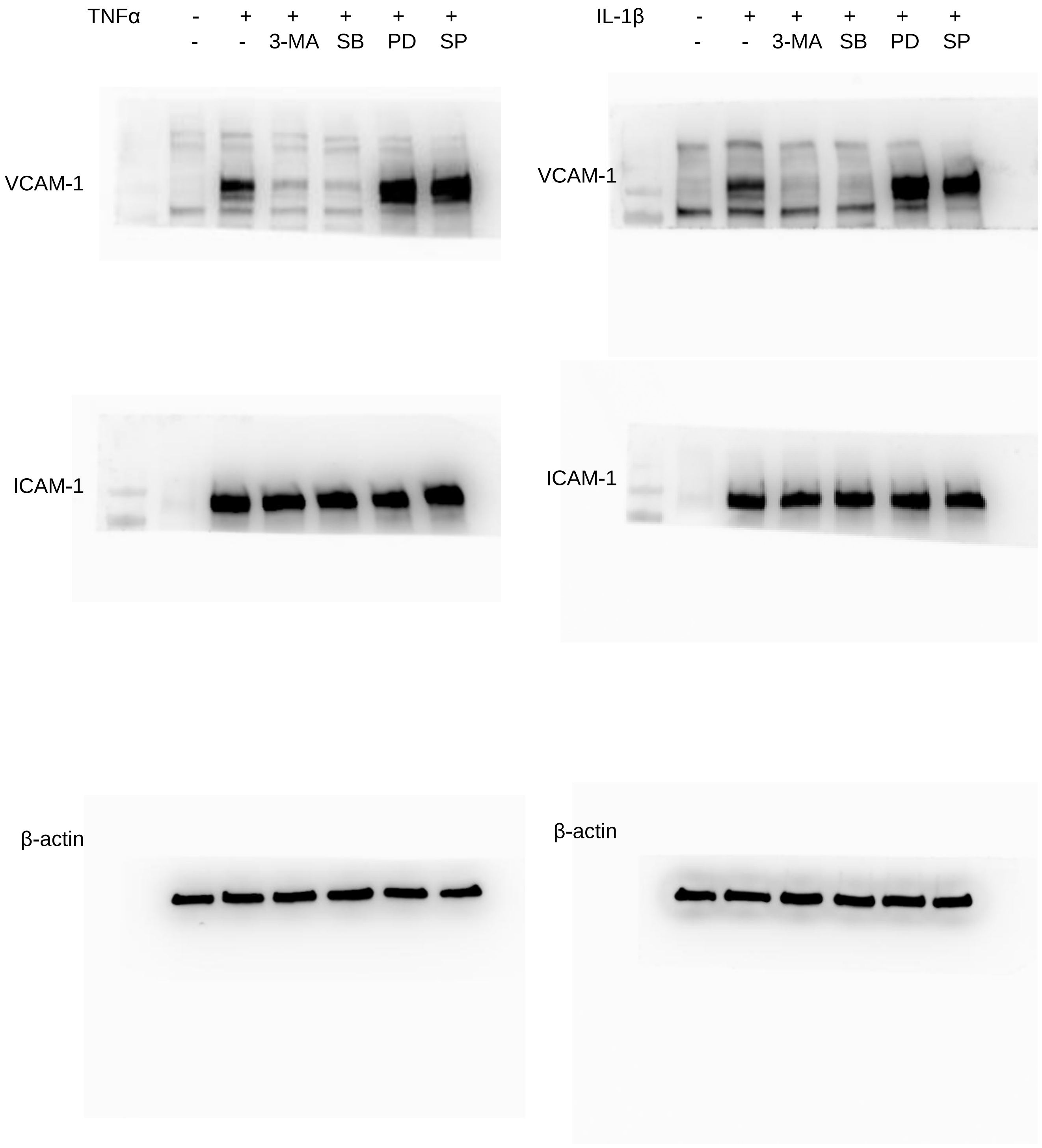

Figure 3. E and F

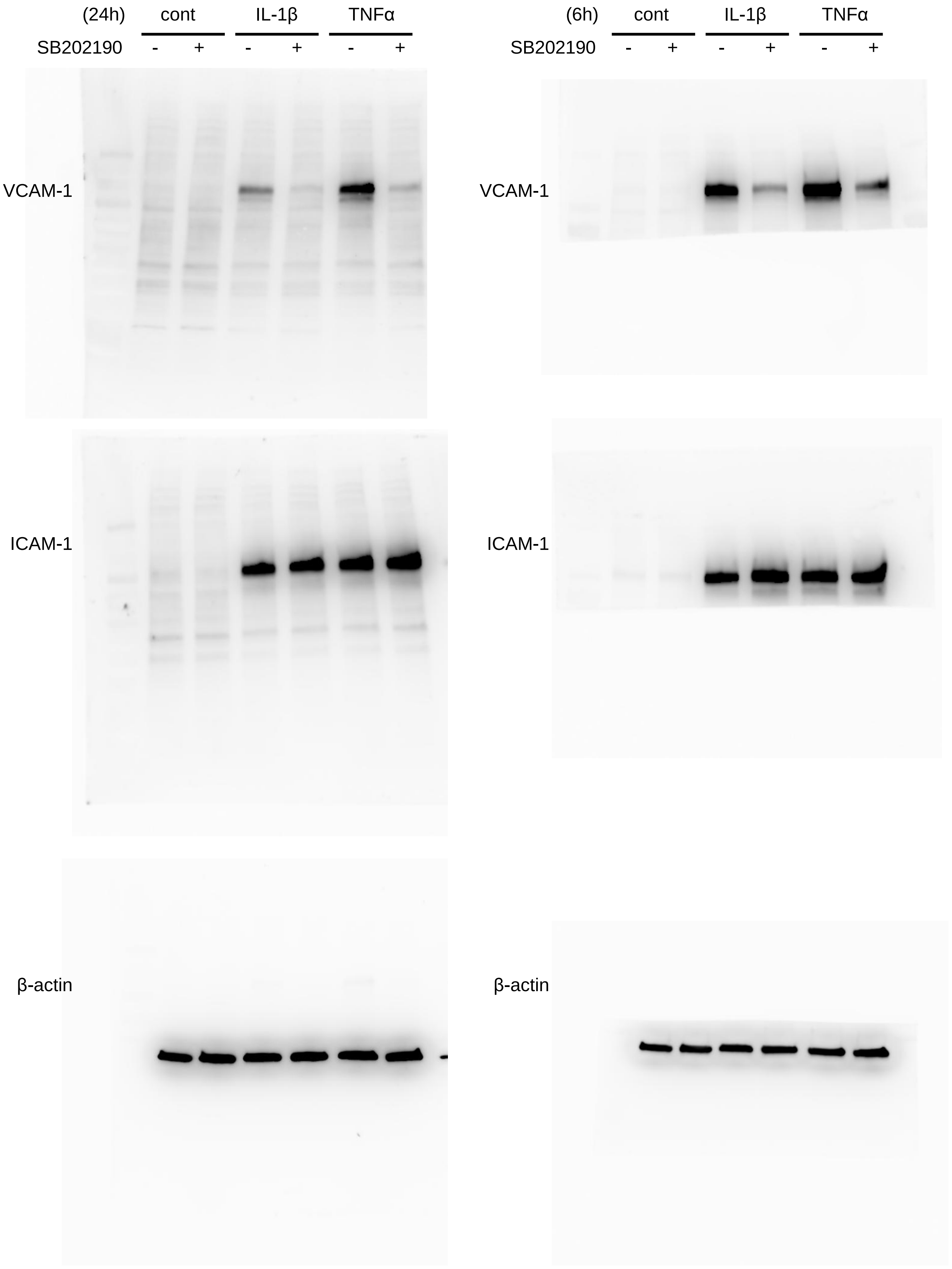

Figure 5. A

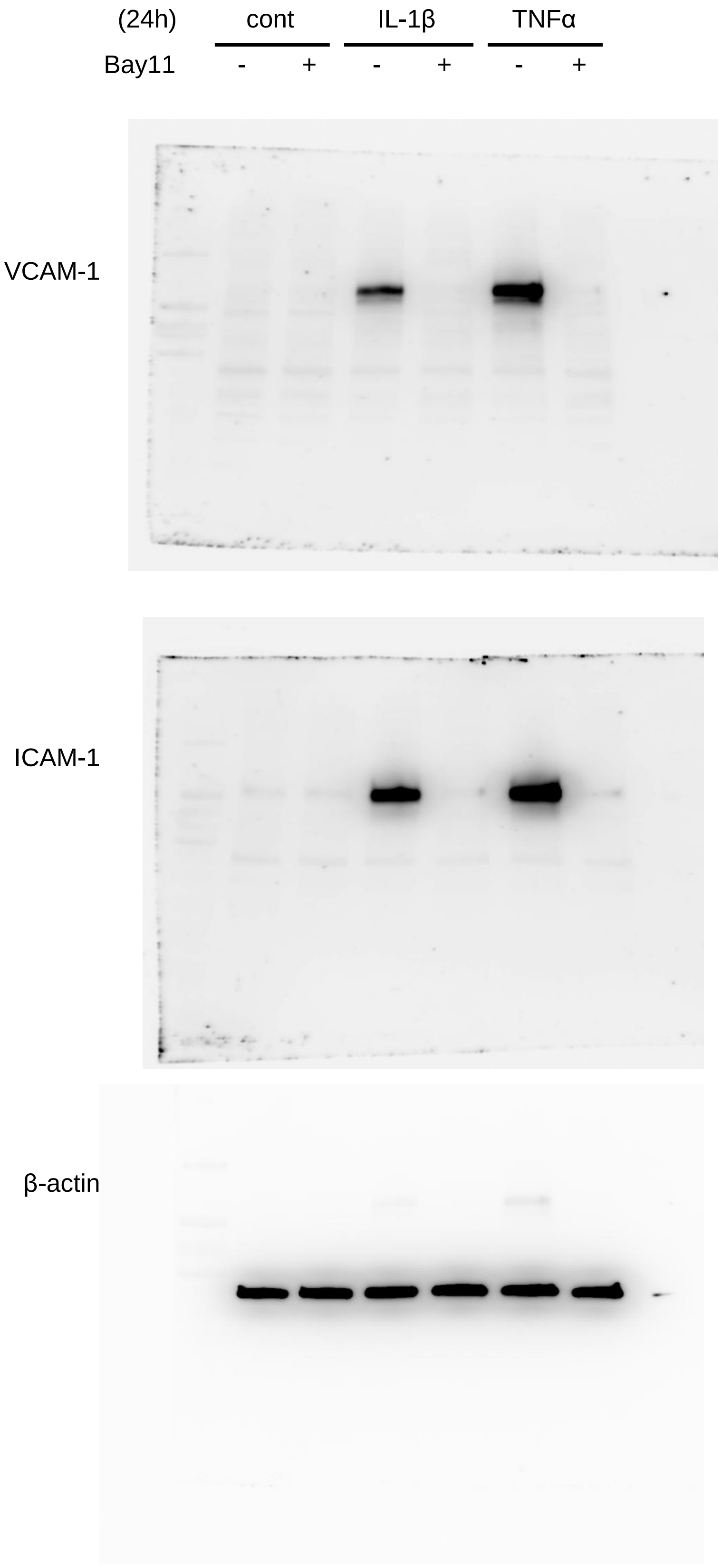

Figure 5. B

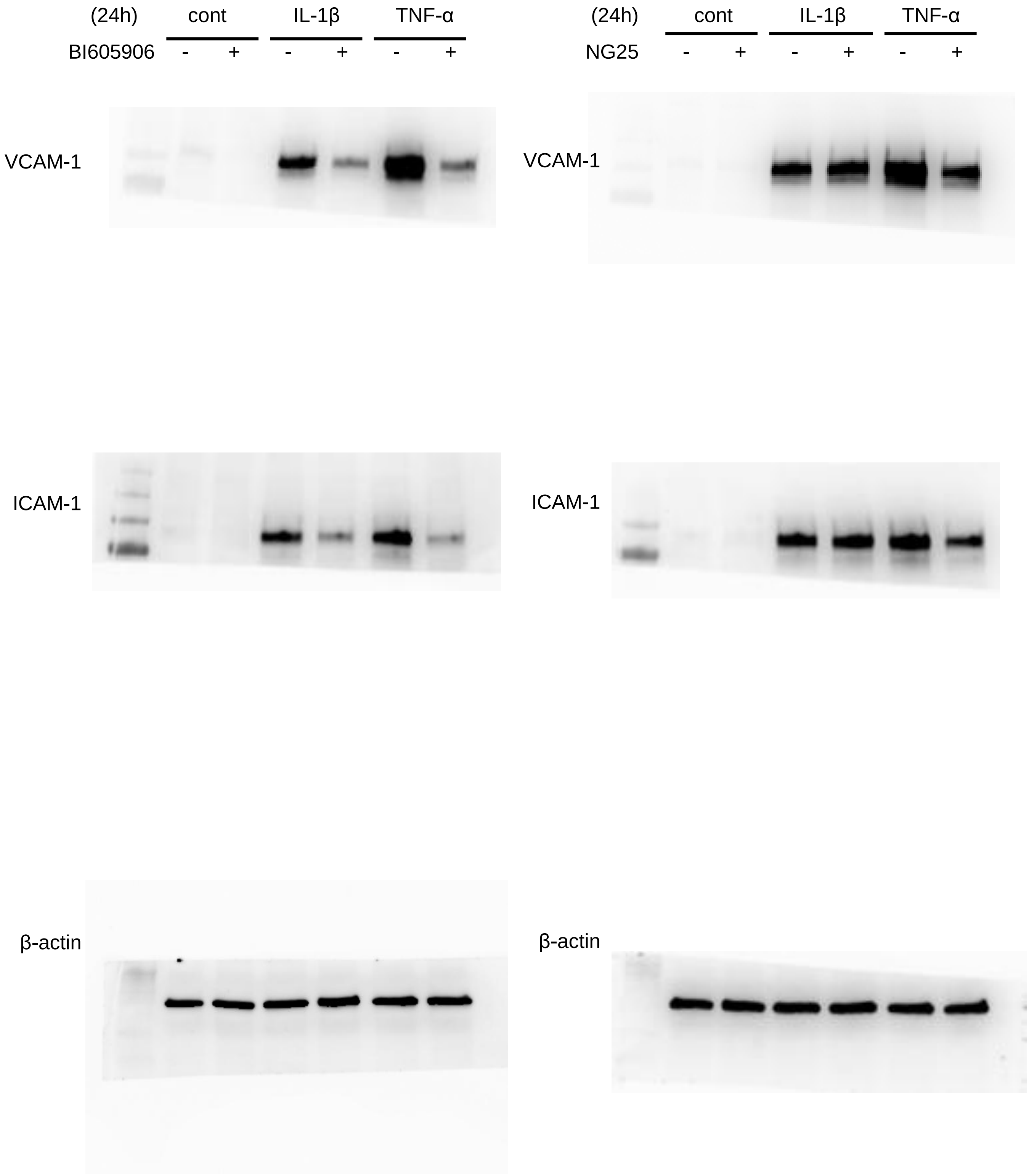
(24h)
cont
IL-1β
TNF-α
BI605906
- + - + - +
VCAM-1
ICAM-1
β-actin
(24h)
cont
IL-1β
TNF-α
NG25
- + - + - +
VCAM-1
ICAM-1
β-actin

Figure 5. C and D nucleus

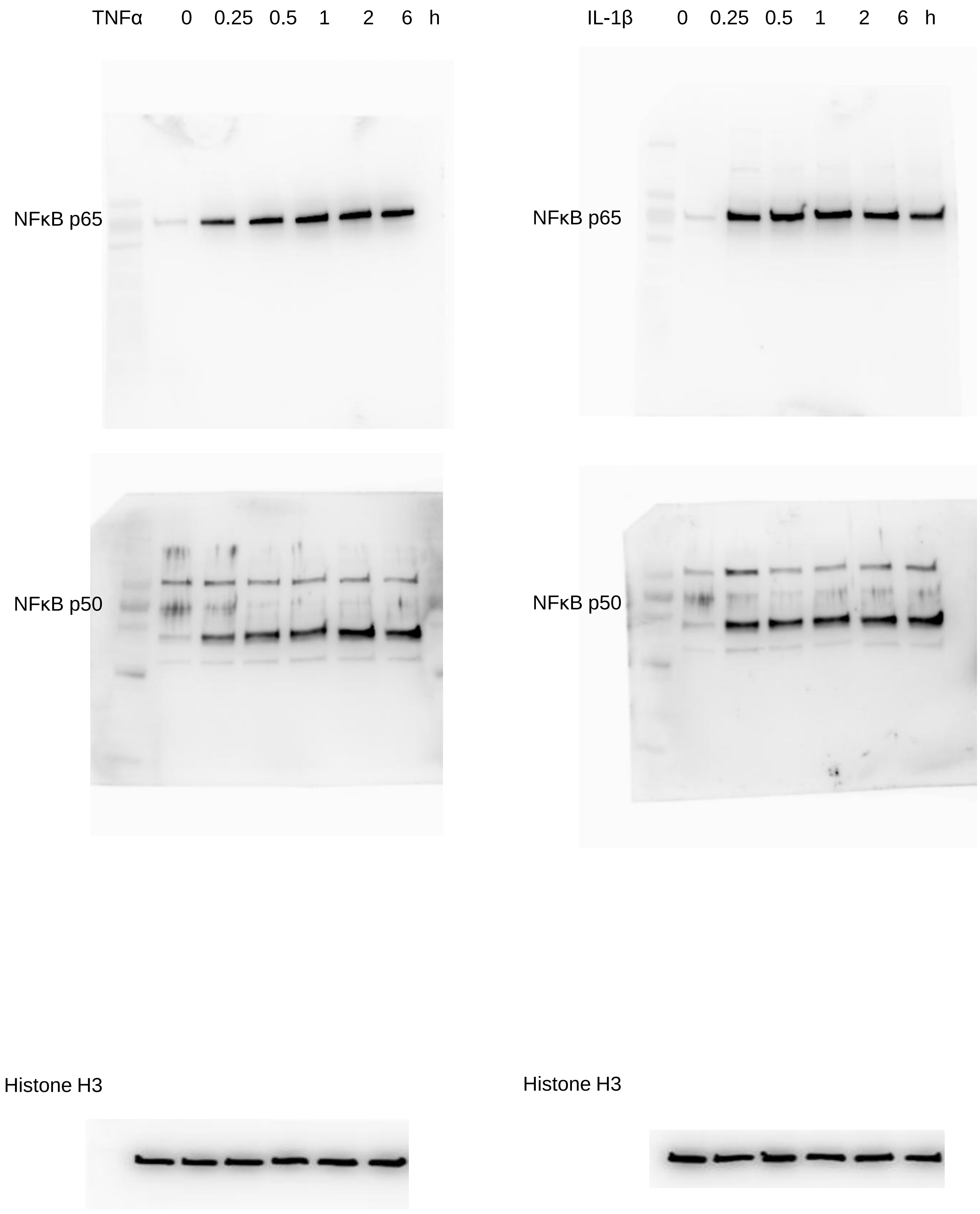

Figure 5. C and D cytosol

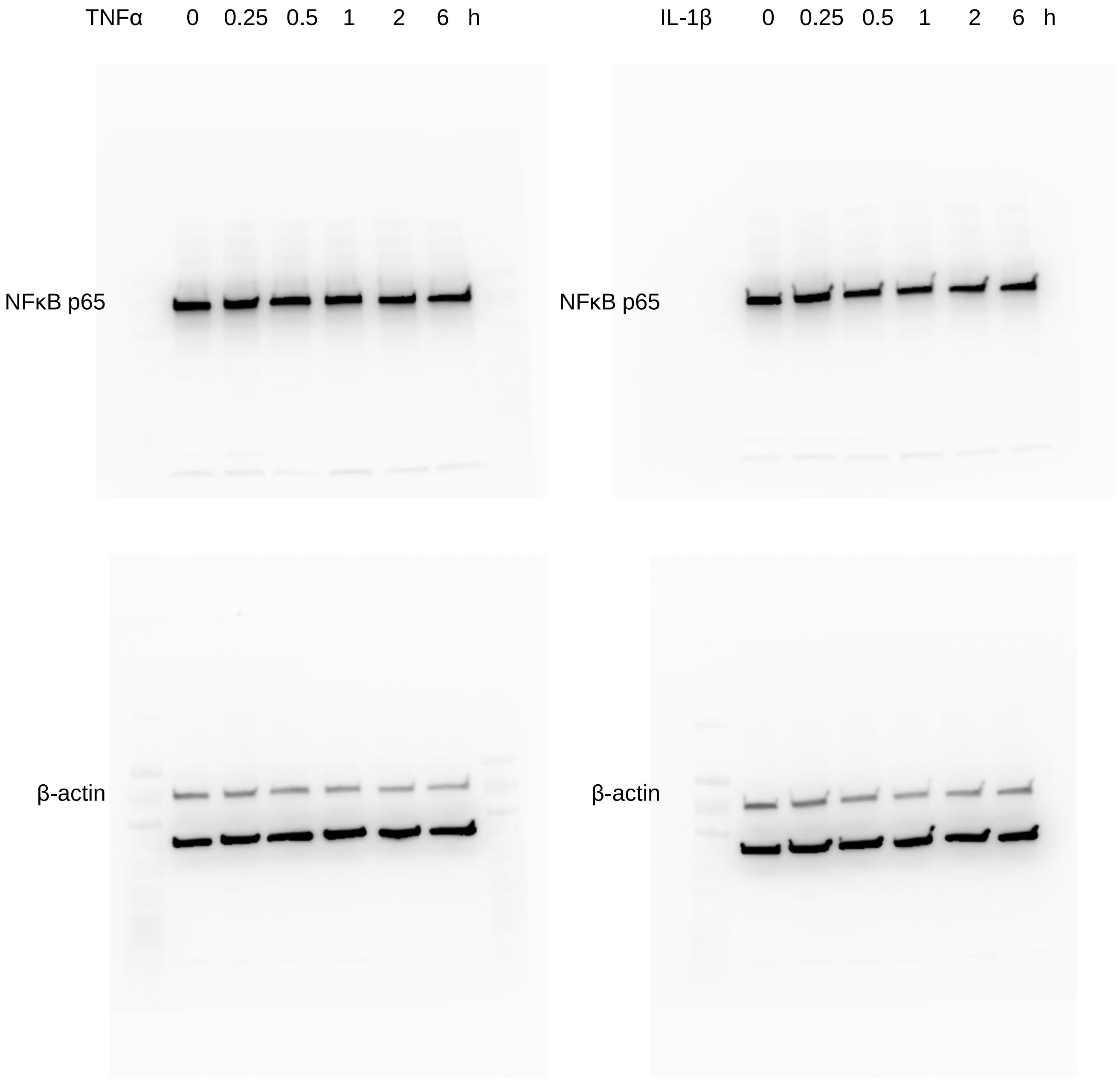

Figure 5. E and F

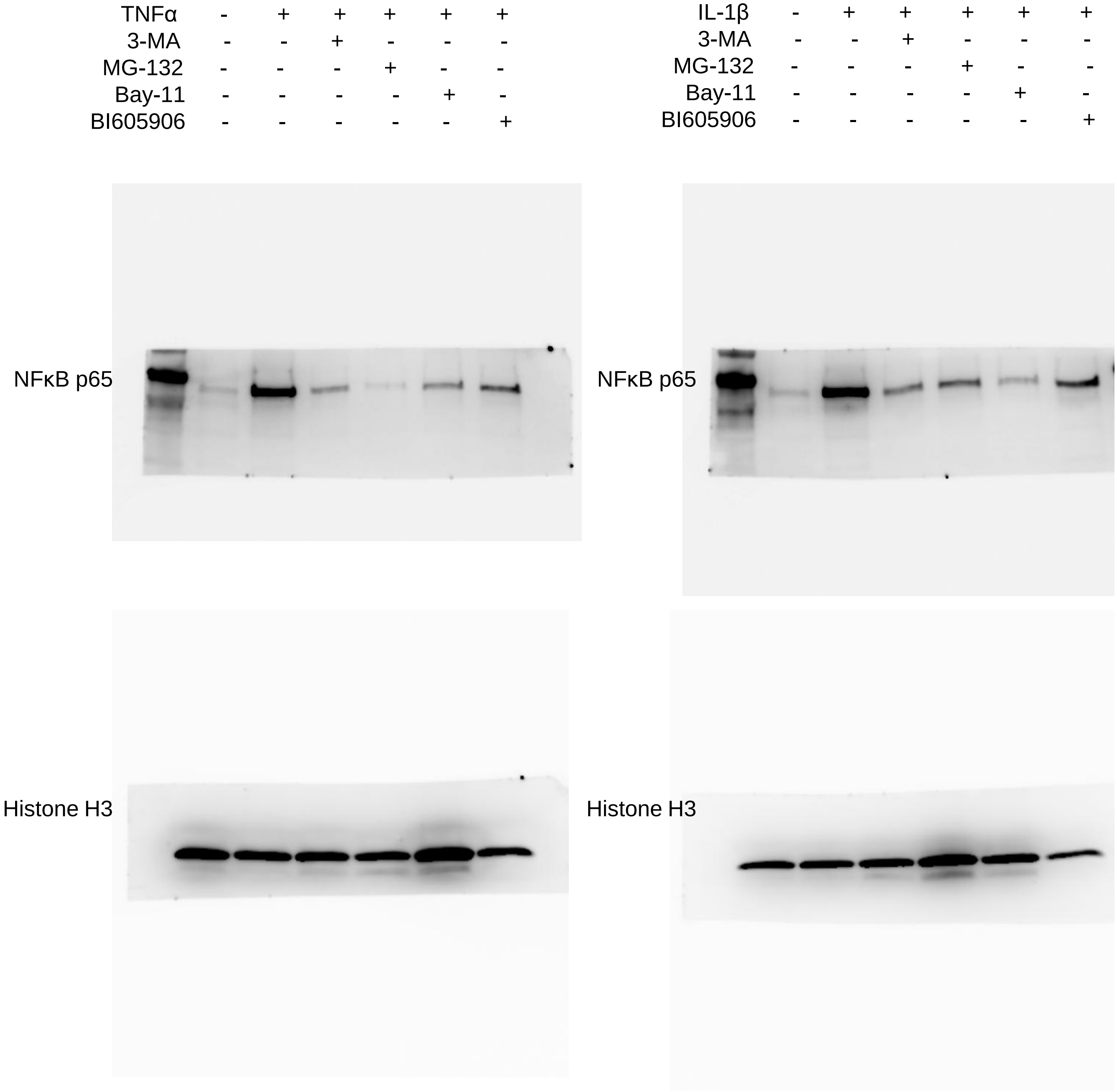

Figure 6. A and B

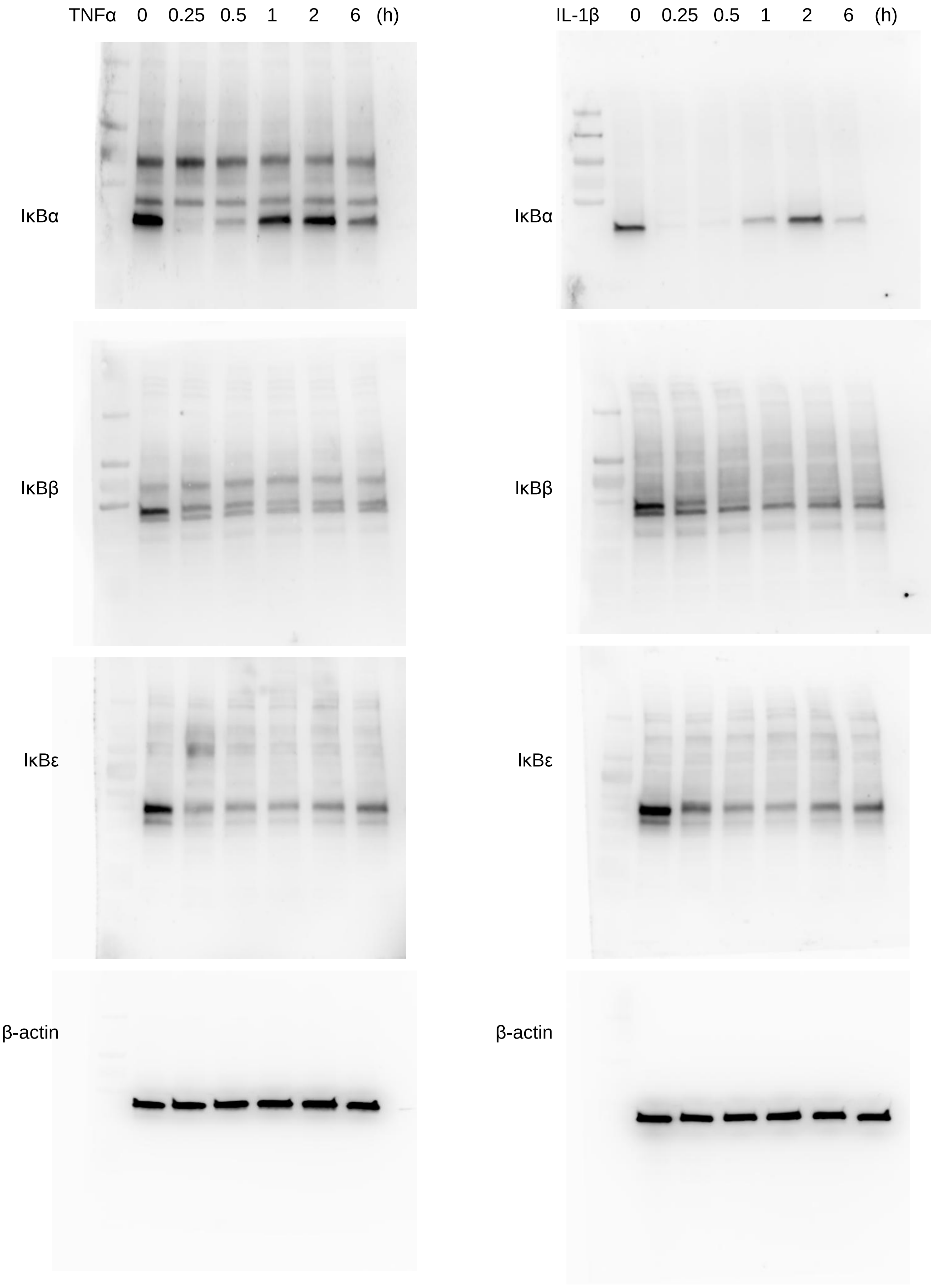

Figure 6. C and D

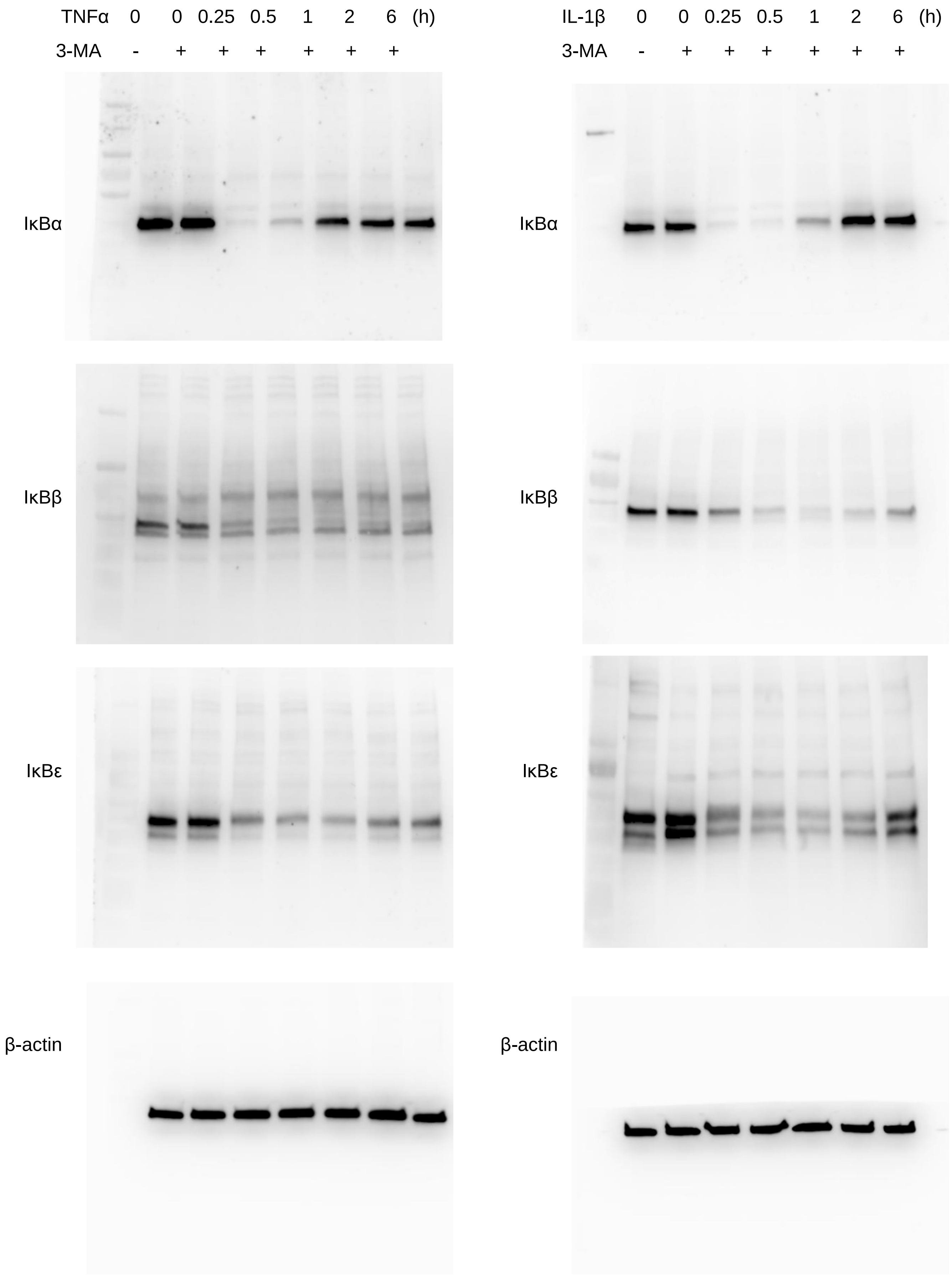

Figure 6. G

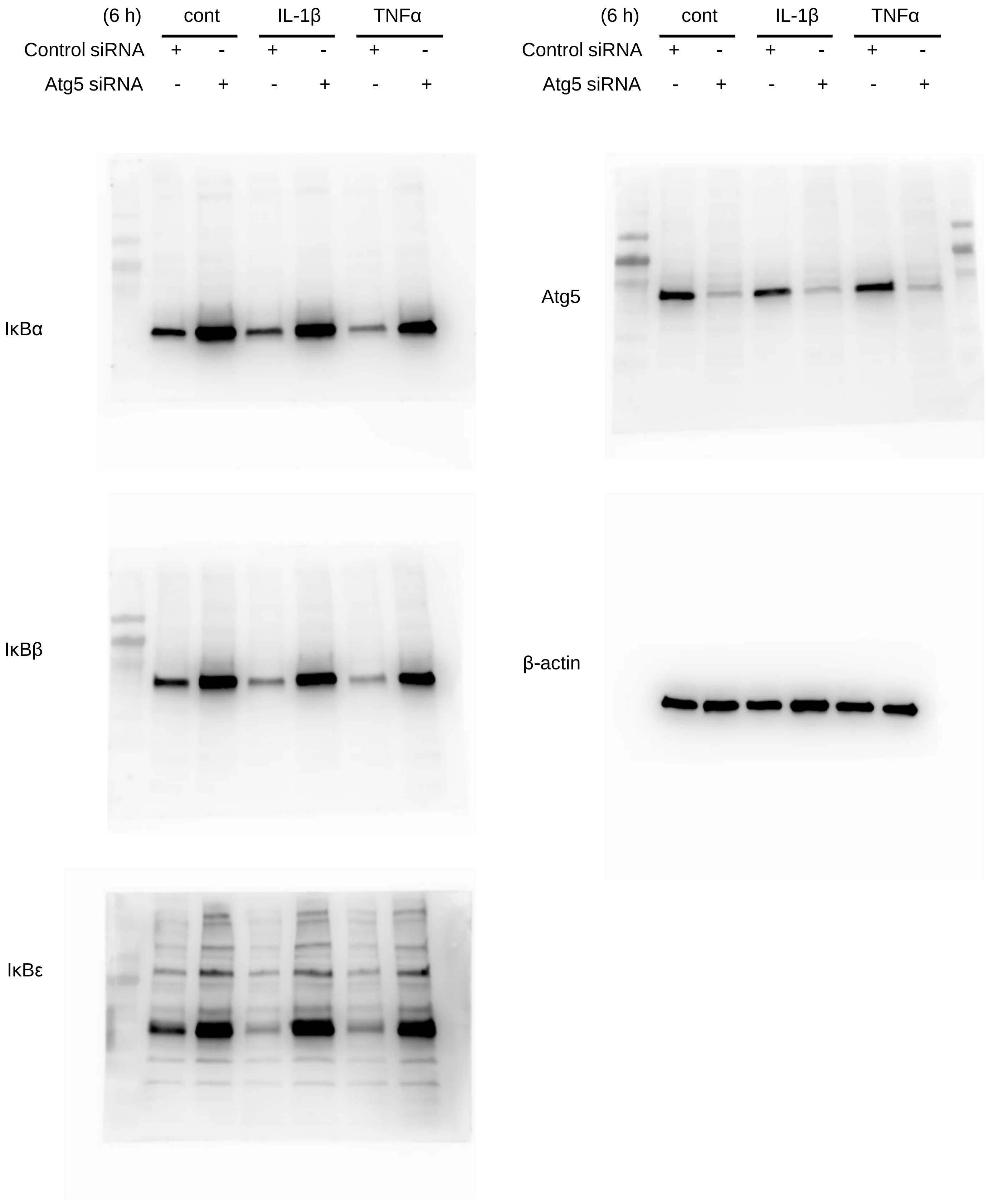
(6 h)
cont
IL-1β
TNFα
Control siRNA + - + - + -
Atg5 siRNA - + - + - +
IκBα
IκBβ
IκBε
(6 h)
cont
IL-1β
TNFα
Control siRNA + - + - + -
Atg5 siRNA - + - + - +
Atg5
β-actin
